# Supplementary material for: A snapshot of mid Eocene landscapes in the southern Central Andes: Spore-pollen records from the Casa Grande Formation (Jujuy, Argentina)
Source: PLoS One. 2023 Apr 5;18(4):e0277389. doi: 10.1371/journal.pone.0277389 (PMC10075436; doi:10.1371/journal.pone.0277389)
Supplement: S1 Appendix — This section includes brief and informal descriptive remarks and dimensions on selected species recorded in the Casa Grande 1 assemblage. (DOCX) [file pone.0277389.s001.docx]

**Supporting information**

**S1 Appendix: Systematic section**

This section includes brief and informal descriptive remarks and dimensions on selected species recorded in the Casa Grande 1 assemblage. Species are listed according to their apertural features within the major plant groups Pteridophytes, Gymnospermophyta, and Magnoliophyta, as in Table 1.

**Monocolpate pollen**

**Genus: *Echimonocolpites* Van der Hammen and García de Mutis 1965**

Type species: *Echimonocolpites ruedae* (Van der Hammen) Van der Hammen and García de Mutis 1965

*Echimonocolpites* sp.

Fig 4N

Description: Pollen grain free, isopolar, elliptical in polar view, monocolpate. Colpi long, extending almost the entire length of the pollen grain, colpi width is not very well defined. Exine 0.8 μm thick, psilate, with spaced (between 0.8-1.3 μm) spines up to 2.6 μm long, some of them with curved ends.

Dimensions: length 21.5 μm, width 10.5 μm, 1 specimen measured.

Comparison: *Echimonocolpites* sp. differs from *E. ruedae* Van der Hammen and García de Mutis 1965 [1] in being smaller and from *E. protofranciscoi* Sarmiento Pérez 1991 [2] in being larger. Moreover, *Echimonocolpites* *panamensis* Jaramillo et al. 2014 [3] is slightly bigger and have acute spines.

Distribution: *Echimonocolpites* species occur from the Early Cretaceus (Valanginian) to the Quaternary [4,5] and extend from America to Africa. In Argentina, there is only one record (*Echimonocolpites* sp.) from the Albian of the Piedra Clavada Formation [6].

Botanical affinity: There is no consensus about the botanical affinity of the species of *Echimonocolpites*. Some of them were referred to palms (e.g. *E*. *coni*, *E. dariensis*, *E. densus*, *E. echiverrucatus*, *E. mauritiformis*, *E. mosquitensis*, *E. pachyexinatus*, *E. panamensis*, *E. protofranciscoi*, *E. ruedae*) [2,3,7,8], while others remain as unknown angiosperms (e.g. *E. acanthus*, *E. echinatus*, *E. tersus*) [9-14].

**Polycolpate pollen**

**Genus: *Nothofagidites* Erdman 1947 ex Potonié 1960**

Type species: *Nothofagidites flemingii* (Couper 1953) Potonié 1960

*Nothofagidites anisoechinatus*  [Menéndez and Caccavari 1975](http://www.fossilworks.org/cgi-bin/bridge.pl?a=displayReference&reference_no=44330&is_real_user=1)

Fig 4O

Description: Pollen grains free, isopolar, peroblate, with straight to slightly convex sides, polycolpate. Colpi equatorial, 6-7, incised c. 3-3.5 μm, colpi margins smooth. Exine 0.7-0.8 μm thick, thinning towards colpi (0.5-0.6 μm thick), stratification obscure. Exine surface densely covered by spines and verrucae approx. 0.5 to 1.1 μm in diameter.

Dimensions: equatorial diameter 23.2-24.6 μm, 4 specimens measured.

Remarks: These pollen grains are similar to the Cretaceus to Oligocene type specimens described by Menéndez and Caccavari [15], particularly in the exine thickness, colpi depth and spines and verrucae size on the exine surface. The specimens described and illustrated by Cornou [16] and Cornou et al. [17] are slightly larger.

Distribution: Specimens recorded from the Casa Grande Formation represent the northernmost record of southern beeches from Argentina. Geographically closest records of *Nothofagidites* came from the Chiquimil Formation (late Miocene from Catamarca, Mautino et al. [18]), YPF.SE. LH.x-2 borehole (Miocene from Santiago del Estero province, Ottone et al., [19]), Cerro Morado Formation (Miocene from La Rioja province, Barreda et al., [20]), Serie del Yeso (Miocene from San Juan province, Barreda et al., [21]), Navidad Formation (early Miocene from Chile, Barreda et al., [22]). Another record came from the Tanque Basin (late Miocene-Pliocene from Brazil, García et al., [23]) but it was considered by Ottone et al. [19] as a doubtful record because the specimen illustrated was poorly preserved and lack diagnostic features of the genus.

Botanical affinity: Nothofagaceae Brassi Type.

*Nothofagidites saraensis* Menéndez and Caccavari 1975

Fig 4P

Description: Pollen grains free, isopolar, peroblate, with convex sides, polycolpate. Colpi equatorial, 5-6, incised c. 2 μm, colpi margins smooth. Exine very thin (approx. 0.7 μm thick), thickening around colpi (1.3 to 1.9 μm thick), stratification obscure. Exine surface sparsely covered by spines approx. 0.7 to 1 μm in diameter.

Dimensions: equatorial diameter 20,1-31.7 μm, 3 specimens measured.

Remarks: These pollen grains are similar to those reported from the Miocene Chenque Formation (eastern Patagonia, Barreda [24,25]) but differ in having a more important thickening around colpi.

Distribution: *Nothofagidites saraensis* is the most common species recorded of the genus and it is commonly found throughout Patagonia and rarely in some localities from northern Argentina (e.g. Santiago del Estero, San Juan, La Rioja, Catamarca, Tucumán) ranging from the late Cretaceous to the Miocene [19,20,21,26,27].

Botanical affinity: Nothofagaceae Fusca Type.

**Colporate pollen**

**Genus: *Baumannipollis* Barreda, 1993**

Type species: *Baumannipollis chubutensis* Barreda, 1993

Species: *Baumannipollis* sp.

Fig 5J

Description: Pollen grains free, isopolar, spheroidal, zonocolporate. Apertures equatorial, 4-8-colporate, colpi short, 8.5 to 15.3 μm in length, and narrow, scarcely surpassing the margin of the ora; ora elliptical, lolongate, endoanulate. Exine stratified, 1.6 to 2.4 μm thick. Subespinal exine approx. 3.2 μm thick. Sexine clearly differentiated, microperforate, thicker than nexine, tectum perforate. Ornamentation echinate, spines solid, 3.5 to 5.5 μm in length, supported on mamelonar bases from 1.6 to 2.8 μm height. Interespinal distance 1.5 to 6.9 μm.

Dimensions: diameter 46.2-59.6 μm without spines, 5 specimens measured.

Comparison: *Baumannipollis* sp. seems to be similar t*o Baumannipollis chubutensis* Barreda 1993 [28] but differs by having a more variable number of apertures.

Botanical affinity: Malvaceae, similar to pollen of extant *Lagunaria*, *Modiolastrum*, *Urocarpidinium* and *Tarasa* [29,30].

**Genus: *Heterocolpites* Van der Hammen 1956 ex Van der Hammen and García de Mutis 1965**

Type species: *Heterocolpites incomptus* Van der Hammen 1956

Comments: The validity of the form-genus *Heterocolpites* and its type species defined by Van der Hammen [31] has long been discussed [32,33]. Yet, the genus was widely used by some authors such as Hoorn [32,34,35]; Jaramillo et al. [3,36-38]; Romero-Baez et al. [39]; da Silva-Caminha et al. [40], and here we decided to accept *Heterocolpites* as a valid genus.

Species: *Heterocolpites rotundus* Hoorn 1993

Fig 5I

Description: Pollen grain free, isopolar, radially symmetric, subprolate, heterocolporate, with 3 colpi alternating with 3 colpori. Subcircular pores 2-3 μm. Exine 1.7 μm columelate, tectate, psilate to slightly scabrate. Nexine and sexine equally thick. Dimensions: polar diameter 22.6 μm, equatorial diameter 19.1 μm, 1 specimen measured.

Remarks: This specimen from Casa Grande Formation has a thicker exine than those from the type material.

Comparison: Although the fossil record of pollen related to Combretaceae-Melastomataceae is widespread in Central America and northern South America, in Argentina there is only one record (‘*Bredeliapollis*’ sp.) from the Oligocene of southern Patagonia [41, 42]. *H. rotundus* is the second fossil pollen record of these families for Argentina, extending their Eocene distribution in the country.

Botanical affinity: Combretaceae-Melastomataceae. Combretaceae and Melastomataceae families include tropical and subtropical plants distributed today mainly in northeastern Argentina, with only one native species (*Terminalia triflora*, Combretaceae) present in the northwestern region, in Yungas jungle [43].

**Genus**: ***Psilatriporites* Van der Hammen 1956 ex. Hoorn 1993**

Type species: *Psilatriporites sarmientoi* Hoorn 1993

Species: *Psilatriporites desilvae* Hoorn 1993

Fig 5N

Description: Pollen grains free, isopolar, subspheroidal, triporate. 3 pseudocolpi long extending to the equatorial margin. Pores in polar view 4.3 to 4.6 μm wide. Exine undifferentiated, relatively thin (approx. 1 μm thick), microreticulate.

Dimensions: equatorial diameter 16.2-19.6 μm, 4 specimens measured.

Comparison: Specimens from Casa Grande Formation are smaller than those of the type material [32].

Botanical affinity: Fabaceae Caesalpinioideae. Members of this subfamily are distributed from tropical and subtropical areas of both hemispheres occupying a wide range of environments like tropical rainforests, evergreen forests, deciduous forests, and savannahs. In South America, the subfamily includes 65 extant genera and 1200 species and most of the genera (30) are endemic to Brazil [44].

**Genus: *Siltaria* Traverse 1955**

Type species: *Siltaria scabriextima* Traverse 1955

Species: *Siltaria dilcheri* da Silva-Caminha et al. 2010

Fig 5B

Description: Pollen grain free, isopolar, subspheroidal, sides convex, tricolporate. Colpi 16.7 to 23 μm length and 2.6 to 4 μm wide, endopores circular, 3.3 μm in diameter. Exine 2.6 μm thick, nexine 0,9 μm thick, sculpture micropitted, lumina 0.5 to 0.6 wide. Dimensions: equatorial diameter 30,6 μm, 1 specimen measured.

Remarks: The specimen from Casa Grande Formation has a slightly thicker exine than those from the type material [40].

Distribution: *Siltaria dilcheri* has been poorly recorded with its first appearance datum (FAD) in the T13 Zone from the Early Miocene of the Solimões Formation [45]. Other occurrences were recorded from the Early Miocene samples of Cucaracha and Culebra formations in Panama [3].

Botanical affinity: Unknown angiosperm.

**Porate pollen**

Comments: There are several genera to assemble periporate pollen grains that differ in structure and number of pores, encompassing several possible botanical affinities, thus fossil genera may share characteristics and affinities. *Psilaperiporites* Regali et al. 1974 [46] includes periporate pollen grains with smooth exine, related to the Chenopodiaceae family. In contrast, *Periporopollenites* Pflug and Thomson (en Thomson and Pflug 1953) [47] assembles periporate specimens with 10 to 32 pores, with or without membrane aperture and punctated-granulated to finely reticulated exine, also related to the Amaranthaceae-Chenopodiaceae complex [48]. Later on Stover and Partridge [49] synonymized the genera *Caryophyllidites* Couper 1960 [50] and *Liquidambarpollenites*  Raatz 1937 ex Potonié 1960 [51] to *Periporopollenites*, and hence widening its botanical affinity to include Caryophyllaceae and Altingiaceae. Finally, Mautino [52] assigned specimens of *Periporopollenites* also to Cactaceae. *Chenopodipollis* Krutzsch 1966 [53] differs from *Periporopollenites* by having a greater number of pores and a thicker exine also assigned to Chenopodiaceae. *Polyporina* Naumova 1939 ex Potonié 1960 [51] also includes periporate forms, with granulate membrane pores and double contour. The genus *Orapollis* Krutzsch 1966 [53] assembles periporate reticulate specimens with one pore per lumen. Muller et al. [4] defined *Maravenites* with the same characteristics of *Orapollis* but with more than 30 pores, leaving the margin from 20 to 30 pores without assignable genus, for this reason Barreda et al. [21] consider *Orapollis* to be periporate, reticulated with one pore in each lumen independent of the total pores number, and the genus *Maravenites* as a synonym of the former. Another genus with similar characteristics is *Gomphrenipollis* Anzótegui and Cuadrado 1996 [48] defined to include periporate, reticulate specimens with the same number of pores as lumens, similar to the extant *Gomphrena*.

The variety of genera and the overlapping characteristics and affinities require special attention, so here we decided to use *Psilaperiporites* to include psilate periporate pollen with uncertain botanical affinity, *Periporopollenites* to periporate with variable ornamentation (non-psilate), and *Gomphrenipollis* to reticulate pollen grains with one pore per lumina similar to *Gomphrena*.

**Genus: *Gomphrenipollis* Anzótegui and Cuadrado 1996**

Type species: *Gomphrenipollis pintadensis* Anzótegui and Cuadrado 1996

Comments: The genus *Gomphrenipollis* was created to include specimens morphologically close to the genus *Gomphrena*. Currently the genus *Gomphrena* has 22 species widely distributed throughout Argentina (with the exception of the provinces of Santa Cruz and Tierra de Fuego) in various biogeographical regions [43]. *Gomphrenipollis* is restricted to the Miocene of Argentina [21]. New contributions included species from the Miocene of Brazil [40,54] and Perú [55]. This finding extends its record to the Eocene and widens the diversity of the taxon.

Species: *Gomphrenipollis* sp. 1

Fig 5S

Description: Pollen grains free, apolar, radially symmetric, spheroidal, pantoporate. About 44 subcircular to circular pores, 3.4-5.2 μm in diameter. Exine 2.5-3.7 μm thick, reticulate, homobrochate. Lumina hexagonal, 7 μm in diameter, walls straight, smooth 1.2 μm width ending in a ridge. In each lumina there is a pore.

Dimensions: diameter 34-37.8 μm, 3 specimens measured.

Comparison: This grain differs from *G. pintadensis* and *Gomphrenipollis* sp. [26] due to its larger size and greater number of pores. *G. garciae* [54] has similar measurements but the original description did not specify the number of pores, also the lumina are polygonal to slightly curved. *G. minimus* [55] is smaller.

Botanical affinity: Amaranthaceae, *Gomphrena*.

Species: *Gomphrenipollis* sp. 2

Fig 5T

Description: Pollen grains free, apolar, radially symmetric, spheroidal, pantoporate. About 18 subcircular to circular pores 3-5.2 μm in diameter. Exine 2-3 μm thick, reticulate, homobrochate, hexagonal lumina 9 μm in diameter, walls straight, smooth ending in a ridge. In each lumina there is a pore.

Dimensions: diameter 25.9-28.9 μm, 3 specimens measured.

Comparison: This morphotype differs from *G. pintadensis* and *Gomphrenipollis* sp. [26] due to its larger size and greater number of pores. *Gomphrenipollis* sp. 1 has more pores. *G. garciae* [54] has similar measurements but the original description did not mention the number of pores, also the lumina are polygonal to slightly curved. *G minimus* [55] is smaller and has more pores.

Remarks: The preservation of the grain was not favorable to observe all characteristics.

Botanical affinity: Amaranthaceae, *Gomphrena*.

**Genus: *Psilaperiporites* Regali et al. 1974**

Type species: *Psilaperiporites robustus* Regali et al. 1974

Species: *Psilaperiporites circinatus* D’Apolito et al. 2021

Fig 5P

Description: Pollen grains free, apolar, radially symmetric, spheroidal, pantoporate. About 20 subcircular to circular pores of different sizes 1.7-5 μm in diameter. Exine 0.7-1 μm, psilate.

Dimensions: diameter 28-35 μm, 6 specimens measured.

Remarks: The pores of different sizes have a regular distribution so that, when the grain collapses, they are superimposed in a circle.

Comments: Similar forms were recorded [56] for the Late Cretaceous of Tanzania [57] and Paleocene-Eocene of Mexico [58], this is the first record for Argentina.

Botanical affinity: Unknown angiosperm.

**Genus: *Periporopollenites* Pflug and Thomson en Thomson and Pflug 1953**

Type species: *Periporopollenites stigmosus* Thomson and Pflug 1953.

Species: *Periporopollenites polyoratus* (Couper, 1960) Stover in Stover and Partridge, 1973

Fig 5R

Description: Pollen grains free, apolar, radially symmetric, spheroidal, pantoporate. About 14 to 15 subcircular pores 1.8-2.6 μm in diameter with granulate membrane. Exine less than 1 μm thick, granulate. Sexine and nexine indistinct.

Dimensions: diameter 20.6-23.7 μm, 2 specimens measured.

Comparison: This grain differs from other described specimens by being smaller. It is very similar to *Polyporina romeroi* [59], but the latter has more spaced pores.

Botanical affinity: Caryophyllaceae-Trimeniaceae. The family Trimeniaceae includes trees and lianas currently distributed in Oceania [60, 61]. Fossils from this family were reported from Japan, Brazil, Australia and Africa [61]. The family Caryophyllaceae includes herbs with a cosmopolitan distribution [61]. In Argentina there are 24 genera and about 112 species [43]. Fossils from this family were reported from Europe, Australia and New Zealand [62]. Both families have a poor fossil record in Argentina [26, 63, 64].

Species: *Periporopollenites* sp.

Fig 5Q

Description: Pollen grains free, apolar, radially symmetric, spheroidal, pantoporate. About 28-30 subcircular to circular pores 3-4 μm in diameter. Exine 2.5-3 μm thick, reticulate, heterobrochate, lumina 0.6-1.2 μm, muri 0.5 μm.

Dimensions: diameter 33.3-34.4 μm, 2 specimens measured.

Comparison: *Periporopollenites* sp. differs from *P. stigmosus* in having a larger number of pores and a thicker reticulated exine with larger lumina. *P. demarcatus* Stover 1973 [49], is tectate and scabrate. *P. vivianae* Mautino 2010 [26], is larger and has a thicker exine. *Periporopollenies* sp. Mautino 2010 [26], is similar in size and number of pores but has a microreticulum, however, the image and affinity are very similar to the specimen found here.

Botanical affinity: Caryophyllaceae, *Silene*.

**Genus: *Pandaniidites* Elsik 1968**

Type species: *Pandaniidites texus* Elsik 1968

Species: *Pandaniidites* sp.

Fig 5L

Description: Pollen grain free, anisopolar, radially symmetric, subspheroidal, monoporate. Circular pore 0.7 μm in diameter, with annulus 0.8 μm wide. Exine about 0.8 μm thick with spines up to 1.4 μm length.

Dimensions: diameter 17.4 μm, 1 specimen measured.

Comparison: *Pandanidites* sp. differs from *P. texus* and *Pandaniidites* sp. from Patagonia (Archangelsky 1973 [59]), by being smaller.

Botanical affinity: Araceae (subfamily Lemnoideae), *Lemna*. Family Araceae comprises tropical herbs with terrestrial, epiphyte or aquatic habit. In Argentina there are four species commonly found in aquatic ecosystems of subtropical areas in northern Argentina. The fossil record of Araceae (leaves, inflorescences and pollen) has been restricted to the Northern Hemisphere. Yet, Gallego et al. [65] recognized that in South America, the Araceae fossil record includes *Pandanidites* pollen grains from Cretaceous and Paleocene sediments from Colombia and Brazil. Subsequently, Cúneo et al. [66] described a community of free floating macrophytes in the Late Cretaceous from Patagonia; in this community they found fossil pollen related to Araceae (*Pandaniidites* sp.). Finally, Farrell et al. [67] recognize another finding for the Miocene of northwestern Argentina. This being a new record of pollen grain related with the subfamily Lemnoideae; in particular the fossil reported here is similar to *L. aequinoctialis* [68].

**References**

1. Van der Hammen T, García de Mutis C. The Paleocene pollen flora of Colombia. Leidse Geologische Mededelingen. 1965; 35(1): 105-114.
2. Sarmiento Pérez G. Palinología de la formación Guaduas. Estratigráfica y sistemática. Boletín Geológico. 1991; 32(1-3): 45-126.
3. Jaramillo CA, Moreno E, Ramírez V, da Silva S, de la Barrera A, de la Barrera A, et al. Palynological record of the last 20 million years in Panama. In: Stevens WD, Montiel OM, Raven P, editors. Paleobotany and biogeography: A festschrift for Alan Graham in his 80th year. St. Louis: Missouri Botanical Garden Press; 2014. p. 134-251.
4. Muller J, de Di Giacomo E, Van Erve AW. A palynological zonation for the Cretaceous, Tertiary, and Quaternary of northern South America. In: American Association Stratigraphic Palynologists Foundation editors. Dallas; 1987.p. 7-76.
5. Brenner GJ, Bickoff IS. Palynology and age of the Lower Cretaceous basal Kurnub Group from the coastal plain to the northern Negev of Israel. Palynology. 1992; 16(1): 137-185.
6. Archangelsky A, Archangelsky S, Poiré D, Canessa N. Registros palinológicos en la Formación Piedra Clavada (Albiano) en su área tipo, provincia de Santa Cruz, Argentina. Revista del Museo Argentino de Ciencias Naturales. Nueva serie. 2008; 10(2): 185-198.
7. Kumaran KN, Edet JJ, Nyong EE. A reappraisal of salviniaceous fossils and its implication on the age and depositional environment of Nkporo Shale of Calabar Flank, Nigeria. Journal of Palaeosciences. 1993; 42(1-3): 210-214.
8. Santos CE. Palynostratigraphy of the Umir Formation, Middle Magdalena Valley Basin (MMVB) Colombia. M. Sc. Thesis, Louisiana State: University and Agricultural and Mechanical College; 2005. Available from: <https://nsgl.gso.uri.edu/lsu/lsuy12012.pdf>.
9. Haag NA. Palinostratigrafia Do Meso-Cenozoico Da Bacia Do Acre (Amazônia Sul Ocidental, Brasil). PHD Thesis, Portugal: Universidade de Coimbra; 2019. Available from: https://www.uc.pt/fctuc/dct/noticias/provas-de-doutoramento-em-geologia-de-nei-a-haag/.
10. de Lima MR, Salard Cheboldaeff M. Palynologie des bassins de Gandarela et Fonseca (Eocene de l'etat de Minas Gerais, Bresil). Boletim IG. 1981; 12: 33-53.
11. Ward JV. Early Cretaceous angiosperm pollen from the Cheyenne and Kiowa formations (Albian) of Kansas, USA. Palaeontographica Abteilung B. 1986; 202(1-6): 1-81.
12. Schrank E. Palynology of the Yesomma Formation in northern Somalia. a study of pollen, spores and associated phytoplankton from the Late Cretaceous Palmae Province. Palaeontographica Abteilung B. 1994; 231: 63-112.
13. Schrank E. Paleoecological aspects of Afropolis/Elaterates peaks (Albian-Cenomanian pollen) in the Cretaceous of Northern Sudan and Egypt. In: Goodman DK, Clarke RT, editors. Proceedings of the IX International Palynological Congress. Dallas: American Association Stratigraphic Palynologists Foundation. AASP; 2001. p. 201-210.
14. Jaramillo CA, Dilcher DL. Middle Paleogene palynology of Central Colombia, South America: a study of pollen and spores from tropical latitudes. Palaeontographica Abteilung B. 2001; 258(4-6): 87-259.
15. Menéndez CA, Caccavari de Filice MA. Distribución y frecuencia del polen fósil de Nothofagus en depósitos Cretácicos y Terciarios del norte de Tierra del Fuego, Argentina. In: Asociación Paleontológica Argentina, editors. Congreso Argentino Paleontología y Bioestratigrafía. Tucumán, 1974. p. 241-256.
16. Cornou ME. Palinoestratigrafía y palinofacies del eoceno medio-oligoceno en la parte central de la Cuenca de Ñirihuau, Argentina. PHD Thesis, Bahía Blanca: Universidad Nacional del Sur; 2012. Available from: https://repositoriodigital.uns.edu.ar/handle/123456789/2268.
17. Cornou ME, Quattrocchio ME, Martínez MA. Palinoestratigrafía de la Formación Salto del Macho, Paleógeno de la Cuenca de Ñirihuau, Argentina. Ameghiniana. 2014; 51(6): 556-571.
18. Mautino LR, Anzótegui LM. Palinología de la Formación Chiquimil (Mioceno Superior), en Río Vallecito, provincia de Catamarca, Argentina. Parte 2. Polen. Ameghiniana. 2002; 39(3): 271-284.
19. Ottone EG, Mazurier SMR, Salinas A. Palinomorfos miocenos del subsuelo de Santiago del Estero, Argentina. Ameghiniana. 2013; 50(5): 509-521.
20. Barreda V, Limarino C, Fauqué L, Tripaldi A, Net L. Primer registro palinológico del miembro inferior de la Formación Cerro Morado (Mioceno), Precordillera de La Rioja. Ameghiniana. 2003; 40(1), 81-87.
21. Barreda VD, Gutiérrez PR, Limarino CO. Edad y paleoambiente de la “Serie del Yeso”, Valle del Cura, Provincia de San Juan: Evidencias palinológicas. Ameghiniana. 1998; 35(3): 321-335.
22. Barreda V, Encinas A, Hinojosa LF. Polen y esporas de la Formación Navidad, Neógeno de Chile. Revista chilena de historia natural. 2011; 84(3), 341-355.
23. Garcia MJ, Bistrichi CA, Saad AR, Campanha VA, Oliveira P. Stratigraphy and palaeoenvironments of the Tanque Basin, southeastern Brazil. Revista Brasileira de Paleontologia. 2008; 11(3), 147-168.
24. Barreda V D. Palinología estratigráfica de las sedimentitas Terciarias del Patagoniano en los alrededores de la ciudad de Comodoro Rivadavia, Provincias de Chubut y Santa Cruz. PHD Thesis, Buenos Aires: Universidad de Buenos Aires; 1989. Available from: https://bibliotecadigital.exactas.uba.ar/download/tesis/tesis_n2282_Barreda.pdf.
25. Barreda VD. Palynomorph assemblage of the Chenque Formation, Late Oligocene?-Miocene from Golfo San Jorge basin, Patagonia, Argentina. Part 3. Polycolpate and tricolporate pollen. Ameghiniana. 1997; 34(2): 131-144.
26. Mautino L R. Palinofloras de las formaciones San José y Chiquimil (Mioceno medio y superior), Noroeste de Argentina. PHD Thesis, Corrientes: Universidad Nacional del Nordeste; 2010.
27. Pujana RR, Fernández DA, Panti C, Caviglia N. The micro-and megafossil record of Nothofagaceae from South America. Botanical Journal of the Linnean Society. 2021; 196(1): 1-20.
28. Barreda VD. Late Oligocene?–Miocene pollen of the families Compositae, Malvaceae and Polygonaceae from the Chenque Formation, Golfo San Jorge basin, southeastern Argentina. Palynology. 1993; 17(1): 169-186.
29. Christensen PB. Pollen morphological studies in the Malvaceae. Grana. 1986; 25(2): 95-117.
30. Cuadrado GA, Miño-Boilini A. Palinología de los géneros Tarasa y Wissadula (Malvaceae, Malveae) de Argentina. Bonplandia. 2006; 15(3-4): 167-187.
31. Van Der Hammen T. Nomenclatura palinológica sistemática. Boletín Geológico. 1956; 4(2-3): 23-62.
32. Hoorn C. Marine incursions and the influence of Andean tectonics on the Miocene depositional history of northwestern Amazonia: results of a palynostratigraphic study. Palaeogeography, Palaeoclimatology, Palaeoecology. 1993; 105(3-4): 267-309.
33. Jansonius J, Hills LV. Genera file of fossil spores. Calgary, Alberta, University of Calgary. Department of Geology, Special Publication. 1976; 3: 287 p.
34. Hoorn C. An environmental reconstruction of the palaeo-Amazon River system (Middle-Late Miocene, NW Amazonia). Palaeogeography, Palaeoclimatology, Palaeoecology. 1994; 112: 187-238.
35. Hoorn C. Fluvial palaeoenvironments in the intracratonic Amazonas Basin (Early Miocene-early Middle Miocene, Colombia). Palaeogeography, Palaeoclimatology, Palaeoecology. 1994; 109: 26-43.
36. Jaramillo CA, Bayona G, Pardo Trujillo A, Rueda M, Torres V, Harrington G, et al. The palynology of the Cerrejón Formation (upper Paleocene) of Northern Colombia. Palynology. 2007; 31(1): 153-189.
37. Jaramillo C, Hoorn C, Silva SAF, Leite F, Herrera F, Quiroz L, et al. The origin of the modern Amazon rainforest: implications of the palynological and palaeobotanical record. In Hoorn C, [Wesselingh](https://www.worldcat.org/es/search?q=au=%22Wesselingh,%20F.%20P.%22) FP. Amazonia, landscape and species evolution: A look into the past. Chichester: Wiley Blackwell; 2010. p. 317-334.
38. Jaramillo CA, Rueda M, Torres V. A palynological zonation for the Cenozoic of the Llanos and Llanos Foothills of Colombia. Palynology. 2011; 35(1): 46-84.
39. Romero Baez M, Silva Caminha S, Leite F, Jaramillo C. Composición y variación en la diversidad florística del Amazonas durante el Neógeno. In: Artabe A; Ballent S, Candela A, Iglesias A, Tortello F, editors. X Congreso Argentino de Paleontología y Bioestratigrafía y VII Congreso Latinoamericano de Paleontología. La Plata. 2010. p. 206.
40. da Silva-Caminha SA, Jaramillo CA, Absy ML. Neogene palynology of the Solimões basin, Brazilian Amazonia. Palaeontographica Abteilung B. 2010; 283(1–3): 13-79.
41. Barreda VD. Palinoestratigrafia de la Formación San Julián en el Área de Playa La Mina (Provincia de Santa Cruz), Oligoceno de la Cuenca Austral. Ameghiniana. 1997; 34(3): 283-294.
42. Palazzesi L., Barreda V. Major vegetation trends in the Tertiary of Patagonia (Argentina): A qualitative paleoclimatic approach based on palynological evidence. Flora. 2007; 202(4): 328-337.
43. Flora Argentina [Internet]. Buenos Aires [cited 2022 Dec 29]. Available from <http://www.floraargentina.edu.ar>.
44. Ulibarri EA. Los géneros de Caesalpinioideae (Leguminosae) presentes en Sudamérica. Darwiniana. 2008; 46(1): 69-163.
45. Gomes BT, Absy ML, D’Apolito C, Caballero-Rodríguez D, Martínez C, Jaramillo C. Miocene paleoenvironments and paleoclimatic reconstructions based on the palynology of the Solimões Formation of Western Amazonia (Brazil). Palynology. 2021; 46(2): 1-19.
46. Regali M da SP, Uesugui N, Santos A da S. Palinologia dos sedimentos meso-cenozóicos do Brasil. Boletim Técnico da Petrobras. 1974; 17: 263-301.
47. Thompson PW, Plug HD. Pollen und Sporen des Mitteleuropäischen Tertiärs. Palaeontographica Abteilung B. 1953; 94:1-138.
48. Anzótegui LM, Cuadrado GA. Palinología de la Formación Palo Pintado, Mioceno Superior, provincia de Salta, República Argentina. Parte 1. Taxones nuevos. Revista Española de Micropaleontología. 1996; 28: 77-92.
49. Stover LE, Partridge AD. Tertiary and Late Cretaceous spores and pollen from the Gippsland Basin, Southeastern Australia. Proc R Soc Victoria. 1973; 85: 237-286.
50. Couper RA. New Zealand Mesozoic and Cainozoic plant microfossils. New Zealand Geological Survey Paleontological Bulletin. 1960; 32: 1-87.
51. Potonié R. Synopsis der Gattungen der Sporae dispersae. III. Teil: Nachträge Sporites, Fortsetzung Pollenites. Beihefte zum Geologischen Jahrbuch. 1960; 39: 1-189.
52. Mautino LR. Nuevas especies de palinomorfos de las Formaciones San José y Chiquimil (Mioceno Medio y Superior) noroeste de Argentina. Especies nuevas. Revista Brasileira de Paleontología. 2011; 14 (3): 279-290.
53. Krutzsch W. Zur Kenntnis der präquartären peripotaten Pollenformen. Geologie. 1966; 55: 16-71.
54. Leite FPR, Silva-Caminha SAFD, D’Apolito C. New Neogene index pollen and spore taxa from the Solimões Basin (western Amazonia), Brazil. Palynology 2021; 45(1): 115-141.
55. Parra FJ, Navarrete RE, di Pasquo MM, Roddaz M, Calderón Y, Baby P. Neogene palynostratigraphic zonation of the Maranon Basin, western Amazonia, Peru. Palynology 2020; 44(4): 675-695.
56. D´Apolito C, Jaramillo C, Harrington G. Miocene palynology of the Solimões Formation (well 1-AS-105-AM), western Brazilian Amazonia. Smithsonian Contributions to Paleobiology. 2021; 105: 1-134.
57. Warny S, Jarzen DM, Haynes SJ, MacLeod KG, Huber BT. Late Cretaceous (Turonian) angiosperm pollen from Tanzania: a glimpse of past vegetation from a warmer climate. Palynology 1998. DOI: 10.1080/01916122.2018.1477850.
58. Smith V, Warny S, Jarzen DM, Demchuk T, Vajda V & Gulick SPS (2019): Paleocene-Eocene palynomorphs from the Chicxulub impact crater, México. Part 2: angiosperm pollen, Palynology (en prensa), DOI: 10.1080/01916122.2019.1705417.
59. Archangelsky S. Palinología del Paleoceno de Chubut. Parte 1: Descripciones Sistemáticas. Ameghiniana. 1973; 10: 339-399.
60. Philipson W.R. Trimeniaceae. In van Steenis C.G.G.J., editor. Flora malesiana. Nijhoff, Dordrecht; 1986. p. 327-333.
61. GROUP TAP (2016) An update of the Angiosperm Phylogeny Group classification for the orders and families of flowering plants: APG IV. Bot. J. Linnean Soc. 181: 1-20.
62. Jordan G.J., Macphail M.K. A middle‐late Eocene inflorescence of Caryophyllaceae from Tasmania, Australia. Am. J. Bot. 2003; 90(5): 761-768.
63. Fernández D.A. Análisis paleoflorístico de la fm Río Turbio (Eoceno de la Provincia de Santa Cruz) y su relación con los cambios paleoclimáticos globales: evidencias esporopolínicas. PHD Thesis, La Plata: Universidad Nacional de La Plata; 2018. Available from: file:///home/macx/Descargas/CONICET_Digital_Nro.5b161f5e-f99e-4cda-bb4d-cd5eb826c586_A-2.pdf
64. Horn M.Y. Palinofloras de las formaciones El Morterito y Palo Pintado (Mioceno superior-Plioceno inferior), Noroeste de Argentina. PHD Thesis, La Plata: Universidad Nacional de La Plata; 2014. Available from: file:///home/macx/Descargas/HORN%202014%20TESIS%20DOCTORAL-2.pdf
65. Gallego J, Gandolfo MA, Cúneo R N, Zamalloa MC. Fossil Araceae from the Upper Cretaceous of Patagonia, Argentina, with implications on the origin of free-floating aquatic aroids. Review of Palaeobotany and Palynology. 2014; 211: 78-86.
66. Cúneo R.N., Gandolfo M.A., Zamalloa M. C., Hermsen E. Late Cretaceous Aquatic Plant World in Patagonia, Argentina. PLoS ONE. 2014; 9(8): 1-18.
67. Farrell E.E., Mautino L.R., Robledo J.M., Palazzesi L. Novedades en la micro y megaflora de la formación Palo Pintado (Mioceno tardío) y su relación con la entomofauna. Libro de resúmenes. XVIII Simposio Argentino de Paleobotánica y Paleontología; sep. 28-30; Jujuy, Argentina. Jujuy: ALPP; 2022. p. 55.
68. Perveen A. A Palynological Survey of Aquatic Flora of Karachi-Pakistan. Tr. J. of Botany. 1999; 23: 309-317.
