# Supplementary material for: A snapshot of mid Eocene landscapes in the southern Central Andes: Spore-pollen records from the Casa Grande Formation (Jujuy, Argentina)
Source: PLoS One. 2023 Apr 5;18(4):e0277389. doi: 10.1371/journal.pone.0277389 (PMC10075436; doi:10.1371/journal.pone.0277389)
Supplement: S1 Table — Frequencies are based on a count of 546 palynomorphs. A black circle indicates frequencies lower than 0.1%. (DOCX) [file pone.0277389.s002.docx]

| **Fossil taxon** | **Botanical affinity** | **%** |
| --- | --- | --- |
| **Trilete spores** | |  |
| *Deltoidospora minor* (Couper) Pocock 1970 | Cyatheaceae, Dicksoniaceae, Schizaeaceae | 0.18 |
| *Leiotriletes* sp. Mautino 2010 | Pteridophyta | 0.37 |
| *Polypodiaceoisporites* cf. *P. retirugatus* Muller 1968 | Pteridaceae (*Pteris*) | 0.18 |
| *Reboulisporites fuegiensis* Zamaloa and Romero 1990 | Ricciaceae | 0.55 |
| *Todisporites minor* Couper 1958 | Osmundaceae | 0.18 |
| *Zlivisporis* sp. | Bryophyta, Hepaticae | 1.47 |
| **Sacate pollen** | |  |
| *Microcachryidites antarcticus* Cookson 1947 | Podocarpaceae (*Microcachrys tetragona*) | ● |
| *Podocarpidites* sp. | Podocarpaceae | 0.37 |
| **Plicate pollen** | |  |
| *Equisetosporites notensis (*Cookson) Romero 1977 | Ephedraceae | ● |
| **Inaperturate pollen** | |  |
| *Smilacipites* cf. *S. herbaceoides* Wodehouse 1933 | Smilacaceae (*Smilax*, *Peltandra*) | ● |
| **Monosulcate pollen** | |  |
| *Arecipites minutiscabratus* (McIntyre 1968) Milne 1988 | Arecaceae | 3.85 |
| *Echimonocolpites* sp. | Arecaceae/unknown angiosperm? | ● |
| *Liliacidites mirus* Srivastava 1969 | Liliaceae | 12.64 |
| *Liliacidites vermireticulatus* Archangelsky and Zamaloa 1986 | Iridaceae | 8.06 |
| *Punctilongisulcites punctiechinatus* (Krutzsch) Casas Gallego and Barrón 2020 | Hydrocharitaceae | 0.55 |
| *Verrumonocolpites* sp. | Unknown angiosperm | ● |
| **Colpate pollen** | |  |
| *Beaupreaidites* sp. | Proteaceae (*Beauprea*) | 1.28 |
| *Nothofagidites anisoechinatus* Menendez and Caccavari 1965 | Nothofagaceae Brassi Type | 0.73 |
| *Nothofagidites saraensis* Menendez and Caccavari 1975 | Nothofagaceae Fusca Type | 0.37 |
| *Retitrescolpites adultus* González Guzmán 1967 | Unknown angiosperm | ● |
| *Tricolpites aspermarginis* McIntyre 1968 | Violaceae | ● |
| *Tricolpites membranus* Couper 1960 | Phytollacaceae (*Seguieria*) | 0.37 |
| *Tricolpites* sp. Mautino 2010 | Oxalidaceae (*Oxalis* spp.) | 1.28 |
| **Colporate pollen** | |  |
| *Ailanthipites* sp. | Anacardiaceae | ● |
| *Baumannipollis* sp. | Malvaceae (*Lagunaria*, *Modiolastrum*, *Urocarpidinium*, *Tarasa*) | 0.37 |
| *Bombacacidites* sp. | Malvaceae Bombacoideae | 1.65 |
| *Foveotricolporites* sp. | Unknown angiosperm | 0.18 |
| *Heterocolpites rotundus* Hoorn 1993 | Combretaceae-Melastomataceae | 0.18 |
| *Malvacipolloides tucumanensis* Mautino et al. 2004 | Malvaceae, Malveae | 0.18 |
| *Margocolporites tenuireticulatus* Barreda 1997 | Fabaceae Mimosoideae | ● |
| *Rhoipites baculatus* Archangelsky 1973 | Fabaceae Papilionoideae (*Aeschynomene*) | 8.97 |
| *Rhoipites guianensis* (Van der Hammen and Wymstra) Jaramillo and Dilcher 2001 | Malvaceae Sterculioideae (*Firmiana* and *Hildegardia*) | 0.37 |
| *Siltaria dilcheri* Silva-Caminha et al. 2010 | Unknown angiosperm | 0.18 |
| **Porate pollen** | |  |
| *Corsinipollenites menendezii* Quattrocchio 1978 | Onagraceae (*Ludwigia*) | ● |
| *Gomphrenipollis* sp. 1 | Amaranthaceae (*Gomphrena*) | 0.37 |
| *Gomphrenipollis* sp. 2 | Amaranthaceae (*Gomphrena*) | 0.73 |
| *Graminidites* sp. | Poaceae | 0.37 |
| *Pandaniidites* sp. | Araceae (*Lemna*) | 0.18 |
| *Periporopollenites polyoratus* (Couper 1960) Stover in Stover and Partridge 1973 | Caryophyllaceae-Trimeniaceae | 0.92 |
| *Periporopollenites* sp. | Caryophyllaceae (*Silene*) | 0.73 |
| *Psilaperiporites circinatus* D’Apolito et al. 2021 | Unknown angiosperm | 1.83 |
| *Psilatriporites desilvae* Hoorn 1993 | Fabaceae Caesalpinioideae | 0.37 |
| *Verrustephanosporites simplex* Leidermeyer 1966 | Ulmaceae (*Phyllostylon*) | 5.49 |
| **Fungal remains** | |  |
| *Biporipsilonites krempii* (Varma and Rawat 1963) Kalgutkar and Jansonius 2000 | Fungi | 0.37 |
| *Colligerites kutchensis* (Kar and Saxena) Jain and Kar 1979 | Fungi | ● |
| *Dictyosporites* sp. Kalgutkar and Braman 2008 | Fungi | 1.47 |
| *Inapertisporites edigeri* Kalgutkar and Jansonius 2000 | Fungi | 0.73 |
| *Inapertisporites subovoideus* (Sheffy and Dilcher) Kalgutkar and Jasonius 2000 | Fungi | 3.66 |
| *Multicellites crassisporus* (Salard-Cheboldaeff and Locquin) Kalgutkar and Jansonius 2000 | Fungi | 2.38 |
| *Pluricellaesporites sheffyi* Martínez Hernández and Tomasini Ortiz 1989 | Fungi | 2.20 |
| *Polycellaesporonites bellus* Chandra et al. 1984 | Fungi | ● |
| *Quilonia* cf. *Q. allepeyensis* (Ramanujam and Rao) Kalgutkar and Jasonius 2000 | Fungi | 0.37 |
| *Reduviasporonites* cf. *R. catenulatus* Wilson 1962 | Fungi | 0.37 |
| *Scolecosporites modicus* Kalgutkar and Jansonius 2000 | Fungi | 0.73 |
| **Zooclasts** | |  |
| Filinia resting eggs | Rotifera, Trochosphaeridae | 0.73 |
| *Hydrozetes* adult leg | Acari, Oribatida | 0.18 |
| Scales of Lepidopteran wings | Lepidoptera | 14.47 |
| Scolecodont-like palynomorphs | Arthropoda? | 2.01 |
| **Other terrestrial palynomorphs** | |  |
| Compact type sclerocyte | - | ● |
| **Fresh-water algae** | |  |
| *Cymatiosphaera* sp. Mays et al. 2021 | Cymatiosphaeraceae | 6.41 |
| *Mougeotia* sp. | Zygnemataceae | ● |
| *Pediastrum biradiatum* Meyen 1829 | Hydrodictyaceae | 0.92 |
| *Pediastrum boryanum* (Turp.) Menegh 1840 | Hydrodictyaceae | 2.56 |
| *Pediastrum duplex* var. *gracillimum* West and West 1895 | Hydrodictyaceae | 1.28 |
| *Planctonites stellarius* (Potonié 1934) Krutzsch 1960 | Zygnematales, Desmidiaceae. | 0.37 |
| *Scenedesmus* sp. | Scenedesmaceae | 1.65 |
| *Spirogyra* sp. 6 Martinez et al. 2008 | Zygnemataceae | 0.18 |
| *Stigmozygodites ministigmosus* Krutzsch and Pacltová 1990 | Zygnemataceae (*Zygnema*) | 0.92 |
| **Marine algae** | |  |
| Acritarch Cyst | Unknwon | 0.18 |
| Dinoflagellate cyst 1 | Dinoflagellata | 0.18 |
| Dinoflagellate cyst 2 | Dinoflagellata | 0.18 |
| Total sum of frequencies |  | 100.00 |

**S3 Table. Identified morphotype list and frequencies (%).** Frequencies are based on a count of 546 palynomorphs. A black circle indicates frequencies lower than 0.1%.
