# Supplementary material for: A snapshot of mid Eocene landscapes in the southern Central Andes: Spore-pollen records from the Casa Grande Formation (Jujuy, Argentina)
Source: PLoS One. 2023 Apr 5;18(4):e0277389. doi: 10.1371/journal.pone.0277389 (PMC10075436; doi:10.1371/journal.pone.0277389)
Supplement: S2 Table — OF: Olmedo Formation, TF: Tunal Formation, MF: Mealla Formation, MGF: Maíz Gordo Formation, LF: Lumbrera Formation, CGF: Casa Grande Formation (this work). (DOCX) [file pone.0277389.s003.docx]

| **Fossil taxon** | **OF** | **TF** | **MF** | **MGF** | **LF** | **CGF** |
| --- | --- | --- | --- | --- | --- | --- |
| *Ailanthipites* spp. | 0 | 0 | 1 | 1 | 0 | 0 |
| *Ailanthipites* sp. | 0 | 0 | 0 | 0 | 0 | 1 |
| *Ailanthipites* sp. Quatrocchio and Volkheimer 1988 | 1 | 1 | 1 | 1 | 0 | 0 |
| *Apiculatisporis* sp. Volkheimer 1972 | 0 | 0 | 1 | 0 | 0 | 0 |
| *Apiculatisporis charahuillaensis* Volkheimer | 1 | 1 | 0 | 0 | 0 | 0 |
| *Araucariacites australis* Cookson 1947 | 0 | 1 | 0 | 0 | 0 | 0 |
| *Araucariacites fissus* Reisser and Williams 1969 | 0 | 1 | 0 | 0 | 0 | 0 |
| *Arecipites minutiscabratus* (McIntyre 1968) Milne 1988 | 0 | 0 | 1 | 0 | 0 | 1 |
| *Azolla sp.* | 0 | 0 | 1 | 1 | 0 | 0 |
| *Baculatisporites* sp. Quattrocchio 1978a | 0 | 0 | 1 | 0 | 1 | 0 |
| *Baumannipollis* sp. | 0 | 0 | 0 | 0 | 0 | 1 |
| *Beaupreaidites* sp. | 0 | 0 | 0 | 0 | 0 | 1 |
| *Biretisporites* sp*.* | 1 | 1 | 1 | 1 | 0 | 0 |
| *Biretisporites* sp. Quattrocchio 1980 | 0 | 0 | 1 | 0 | 0 | 0 |
| *Bombacacidites* sp*.* | 0 | 0 | 0 | 0 | 0 | 1 |
| Indeterminate cingulate spore 1 | 0 | 1 | 0 | 0 | 0 | 0 |
| Indeterminate cingulate spore 2 | 0 | 1 | 0 | 0 | 0 | 0 |
| *Cingulatisporites* sp. | 0 | 1 | 0 | 0 | 0 | 0 |
| *Cingutriletes australis* Archangelsky 1972 | 0 | 0 | 0 | 0 | 0 | 1 |
| *Classopollis* sp. | 0 | 0 | 0 | 1 | 0 | 0 |
| *Clavatricolpites gracilis* González Guzmán 1967 | 0 | 1 | 1 | 1 | 0 | 0 |
| *Corsinipollenites menendezii* (Quattrocchio) Volkheimer et al. 1984 | 1 | 1 | 1 | 1 | 1 | 1 |
| *Cricotriporites guianensis* Leidelmeyer, 1966 | 0 | 0 | 0 | 1 | 0 | 0 |
| *Cricotriporites* sp. A | 0 | 0 | 0 | 1 | 0 | 0 |
| *Cyathidites patagonicus* Archangelsky 1972 | 1 | 0 | 0 | 0 | 0 | 0 |
| *Cycluspahera psilata* Volkheimer and Sepúlveda 1976 | 1 | 1 | 0 | 0 | 0 | 0 |
| *Deltoidospora minor* (Couper) Pocock 1970 | 0 | 1 | 0 | 0 | 0 | 1 |
| *Deltoidospora neddeni* Pflug in Thomson and Pflug 1953 | 0 | 1 | 0 | 0 | 0 | 0 |
| *Deltoidospora* sp*.* | 0 | 0 | 1 | 0 | 0 | 0 |
| *Deltoidospora* sp. Leanza et al. 2002 | 0 | 0 | 0 | 0 | 0 | 1 |
| *Dictyophyllidites* sp. | 0 | 0 | 0 | 1 | 0 | 0 |
| *Echistephanoporites alfonsí* Leidelmeyer 1966. | 0 | 0 | 0 | 1 | 0 | 0 |
| *Ephedripites multicostatus* Brenner 1963 | 1 | 1 | 0 | 0 | 0 | 0 |
| *Equisetosporites* notensis | 0 | 0 | 0 | 0 | 0 | 1 |
| *Ephedripites* cf. *E.* sp. 1 of Frederiksen et al. (1983) sensu Quattrocchio and Volkheimer (1988) | 0 | 1 | 1 | 1 | 0 | 0 |
| *Foveotricolporites* sp. | 0 | 0 | 0 | 0 | 0 | 1 |
| *Foveotriletes* sp. | 1 | 0 | 0 | 0 | 0 | 0 |
| *Gabonisporis vigorouxii* Boltenhagen 1967 | 1 | 1 | 0 | 0 | 1 | 0 |
| *Gemmatricolpites subsphaericus* Archangelsky 1973 | 0 | 1 | 1 | 1 | 0 | 0 |
| *Gleicheniidites argentinus* Volkheimer 1972 | 0 | 1 | 0 | 0 | 0 | 0 |
| *Gomphrenipollis* sp. 1 | 0 | 0 | 0 | 0 | 0 | 1 |
| *Gomphrenipollis* sp. 2 | 0 | 0 | 0 | 0 | 0 | 1 |
| *Graminidites* sp. | 0 | 0 | 0 | 1 | 0 | 1 |
| *Grapnelispora evansii* Stover and Partridge 1984 | 0 | 0 | 1 | 0 | 0 | 0 |
| *Heterocolpites rotundus* | 0 | 0 | 0 | 0 | 0 | 1 |
| *Inaperturopollenites* sp. D Quatrocchio 1980 | 0 | 1 | 0 | 1 | 1 | 0 |
| *Inaperturopollenites* sp. E Quatrocchio 1978c | 0 | 0 | 0 | 0 | 1 | 0 |
| *Inaperturopollenites* spp. | 0 | 0 | 0 | 1 | 0 | 0 |
| *Ischyosporites* spp. | 0 | 0 | 1 | 0 | 0 | 0 |
| *Laevigatosporites* sp. A Quattrocchio 1978c | 0 | 0 | 0 | 0 | 1 | 0 |
| *Leiotriletes* sp. Mautino 2010 | 0 | 0 | 0 | 0 | 0 | 1 |
| *Leptolepidites macroverrucosus* Schulz 1967 | 1 | 0 | 0 | 0 | 0 | 0 |
| *Liliacidites mirus* | 0 | 0 | 0 | 0 | 0 | 1 |
| *Liliacidites variegatus* Couper 1953 | 0 | 0 | 1 | 0 | 0 | 0 |
| *Liliacidites vermireticulatus* Archangelsky and Zamaloa 1986 | 0 | 0 | 0 | 0 | 0 | 1 |
| *Liquidambarpollenites brandonensis* Traverse 1955  sensu Quattrocchio 1978b | 0 | 0 | 1 | 0 | 1 | 0 |
| *Malvacipolloides tucumanensis* | 0 | 0 | 0 | 0 | 0 | 1 |
| *Margocolporites tenuireticulatus* | 0 | 0 | 0 | 0 | 0 | 1 |
| *Microcachryidites antarcticus* Cookson 1947 | 0 | 0 | 0 | 0 | 0 | 1 |
| *Mtchedlishvilia saltenia* Moroni 1984 | 1 | 1 | 0 | 0 | 0 | 0 |
| *Myriophyllumpollenites* spp. | 0 | 0 | 1 | 1 | 0 | 0 |
| *Myriophyllumpollenites* sp. 1 | 1 | 1 | 0 | 0 | 0 | 0 |
| *Myriophyllumpollenites* sp. 2 | 0 | 1 | 0 | 0 | 0 | 0 |
| *Nothofagidites anisoechinatus* Menendez and Caccavari 1965 | 0 | 0 | 0 | 0 | 0 | 1 |
| *Nothofagidites saraensis* Menendez and Caccavari 1975 | 0 | 0 | 0 | 0 | 0 | 1 |
| *Nothopollenites* sp. | 0 | 0 | 0 | 0 | 1 | 0 |
| *Pandaniidites texus* Elsik 1968 | 1 | 1 | 1 | 0 | 0 | 0 |
| *Pandaniidites* sp. Archangelsky 1973 | 0 | 0 | 1 | 1 | 0 | 1 |
| *Peninsulapollis gilli* (Cookson) Dettmann y Jarzen 1988 | 1 | 0 | 0 | 0 | 0 | 0 |
| *Periporopollenites polyoratus* | 0 | 0 | 0 | 0 | 0 | 1 |
| *Periporopollenites* sp. | 0 | 0 | 0 | 0 | 0 | 1 |
| *Podocarpidites marwickii* Couper 1953 | 0 | 1 | 0 | 1 | 1 | 0 |
| *Podocarpidites* sp. | 1 | 0 | 0 | 0 | 0 | 1 |
| *Polypodeaceoisporites retirugatus* Muller 1968 | 0 | 1 | 0 | 0 | 0 | 1 |
| *Psilaperiporites circinatus* D’Apolito et al. 2021 | 0 | 0 | 0 | 0 | 0 | 1 |
| *Psilatricolpites acerbus* González Guzmán 1967 | 0 | 0 | 1 | 0 | 0 | 0 |
| *Psilatricolpites inargutus* (McIntyre) Archangelsky 1973 | 1 | 0 | 0 | 1 | 0 | 0 |
| *Psilatricolpites salamanquensis* Archangelsky y Zamaloa 1986 | 0 | 0 | 1 | 0 | 0 | 0 |
| *Psilatricolpites simplex* González Guzmán 1967 | 0 | 0 | 1 | 0 | 0 | 0 |
| *Psilatricolpites* sp. | 0 | 0 | 0 | 1 | 0 | 0 |
| *Psilatriporites deslivae* Hoorn 1993 | 0 | 0 | 0 | 0 | 0 | 1 |
| *Puntilongisulcites puntiechinatus* (Krutzsch) Casas Gallego and Barrón 2020 | 0 | 0 | 0 | 0 | 0 | 1 |
| *Quillembaypollis* sp. | 0 | 0 | 0 | 0 | 0 | 1 |
| *Reboulisporites fuegiensis* Zamaloa and Romero 1990 | 0 | 0 | 0 | 0 | 0 | 1 |
| *Restioniidites* spp. | 0 | 0 | 1 | 0 | 0 | 0 |
| *Retitricolporites chubutensis* Archangelsky 1973 | 0 | 1 | 1 | 1 | 0 | 0 |
| *Retitricolporites medius* González Guzmán 1967 | 0 | 0 | 0 | 1 | 0 | 0 |
| *Retitricolporites* sp. A | 0 | 1 | 0 | 0 | 0 | 0 |
| *Retitriletes austroclavatidites* (Cookson) Döring et al. en Krutzsch 1963 | 1 | 1 | 0 | 1 | 1 | 0 |
| *Rhoipites baculatus* Archangelsky 1973 | 1 | 1 | 1 | 1 | 0 | 1 |
| *Rhoipites guianensis* (Van der Hammen and Wymstra) Jaramillo and Dilcher 2001 | 0 | 0 | 0 | 0 | 0 | 1 |
| *Rhoipites minusculus* Archangelsky, 1973 | 0 | 1 | 1 | 1 | 0 | 0 |
| *? Rhoipites* sp. A of Quattrocchio 1978a | 0 | 1 | 1 | 1 | 1 | 0 |
| *Rhoipites* sp. A Quattrocchio and Volkheimer 1990 | 0 | 1 | 0 | 0 | 0 | 0 |
| *Rhoipites* sp. B of Quattrocchio et al. 1988 | 1 | 1 | 1 | 0 | 0 | 0 |
| *Rhoipites* sp. B Quatrocchio and Volkheimer | 1 | 1 | 0 | 0 | 0 | 0 |
| *Rhoipites* sp. | 0 | 0 | 0 | 1 | 0 | 0 |
| *Rhoipites* sp. 1 | 1 | 0 | 0 | 0 | 0 | 0 |
| *Rhoipites* sp. 2 | 1 | 0 | 0 | 0 | 0 | 0 |
| *Rousea patagónica* Archangelsky 1973 | 1 | 1 | 1 | 1 | 0 | 0 |
| *Rugulatisporites* sp. | 0 | 1 | 0 | 0 | 0 | 0 |
| *Rugumonoporites* sp. A | 0 | 1 | 0 | 0 | 0 | 0 |
| *Siltaria dilcheri* Silva-Caminha et al. 2010 | 0 | 0 | 0 | 0 | 0 | 1 |
| *Smilacipites* cf. *S. herbaceoides* Wodehouse 1933 | 0 | 0 | 0 | 0 | 0 | 1 |
| *Smilacipites saltensis* Quattrocchio 1978a | 0 | 0 | 0 | 1 | 1 | 0 |
| *Smilacipites* sp*.* | 0 | 0 | 0 | 0 | 1 | 0 |
| *Spinitricolpites jennerclarkei* Scholtz ex Jansonius and Hills 1990 | 1 | 0 | 0 | 0 | 0 | 0 |
| *Spinizonocolpites* sp. | 1 | 1 | 1 | 1 | 0 | 0 |
| *Spinizonocolpites* sp. Archangelsky 1973 | 0 | 0 | 1 | 1 | 0 | 0 |
| *Srivastavapollenites exoticus* Ruiz and Quattrocchio 1993 | 1 | 0 | 0 | 0 | 0 | 0 |
| *Striatricolporites* spp. | 0 | 0 | 0 | 1 | 0 | 0 |
| *Todisporites minor* Couper 1958 | 0 | 0 | 0 | 0 | 0 | 1 |
| *Tricolpites asperamarginis* McIntyre 1968 | 0 | 0 | 0 | 0 | 0 | 1 |
| *Tricolpites bibaculatus* Archangelsky and Zamaloa 1966 | 0 | 0 | 1 | 0 | 0 | 0 |
| *Tricolpites communis* Archangelsky 1973 | 0 | 0 | 1 | 1 | 1 | 0 |
| *Tricolpites (Psilatricolpites) lumbrerensis* Quattrocchio 1980 | 1 | 1 | 0 | 0 | 1 | 0 |
| *Tricolpites membranus* Couper 1960 | 0 | 0 | 0 | 0 | 0 | 1 |
| *Tricolpites reticulatus* Cookson 1947 | 1 | 1 | 1 | 1 | 1 | 0 |
| *Tricolpites trioblatus* Mildenhall and Pocknall 1989 | 1 | 0 | 0 | 0 | 0 | 0 |
| *Tricolpites vulgaris* Pierce 1961 | 0 | 1 | 0 | 0 | 1 | 0 |
| *Tricolpites* sp. Mautino 2010 | 0 | 0 | 0 | 0 | 0 | 1 |
| *Tricolpites* sp. A | 0 | 1 | 0 | 0 | 1 | 0 |
| *Triorites* sp. Quatrocchio and Salfity 1984 | 0 | 0 | 0 | 1 | 0 | 0 |
| *Verrucosisporites* sp. | 0 | 1 | 0 | 0 | 0 | 0 |
| *Verrumonocolpites* sp. | 0 | 0 | 0 | 0 | 0 | 1 |
| *Verrustephanoporites simplex* Leidelmeyer 1966 | 1 | 1 | 1 | 1 | 1 | 1 |
| *Zlivisporis* sp. | 0 | 0 | 0 | 0 | 0 | 1 |
| *Zlivisporis* sp. 1 | 1 | 1 | 0 | 0 | 0 | 0 |
| *Zlivisporis* sp. 2 | 1 | 1 | 0 | 0 | 0 | 0 |

**References:**

Narváez PL. Palinoestratigrafía, paleoambientes y cambios climáticos durante el Cretácico final y Paleógeno de la Cuenca del grupo Salta, República Argentina. [Ph.D. thesis]. Mendoza: Universidad Nacional de Cuyo; 2009. Available from: <https://planificacion.bdigital.uncu.edu.ar/objetos_digitales/5502/narvaez-tesisd.pdf>

Narváez PL, Volkheimer W. Nuevos datos palinológicos de la Formación Tunal, Daniano de la Cuenca del Grupo Salta. In: de Barrio RE, Etcheverry RO, Caballé MF, Llambías EJ, editors. 16° Congreso Geológico Argentino; 2005 Sep, La Plata, Argentina. Buenos Aires: Asociación Geológica Argentina, 2005. p. 465-466.

Narvaez P, Volkheimer W. Palynostratigraphy and paleoclimatic inferences of the Balbuena and Santa Bárbara subgroups (Salta Group Basin, Cretaceous–Paleogene): Correlation with Patagonian basins. Cenozoic geology of the Central Andes of Argentina 2011: 283-300.

Quatrocchio, M. Estudio palinológico preliminar de la Formación Lumbrera (Grupo Salta), localidad Pampa Grande, Provincia de Salta, República de Argentina. In: Bertels A, Romero EJ, Baez AM, Cione A, Caccavari de Felice M, Ganduglia P, et al., editors. II Congreso Argentino de Paleontologıa y Biostratigrafıa, I Congreso Latinoamericano de Paleontologıa; 1978 Apr 2-6; Buenos Aires, Argentina. Buenos Aires: Asociación Paleontológica Argentina; 1980. p. 131-149.

Quattrocchio M. Contribución al conocimiento de la palinología estratigráfica de la Formación Lumbrera (Terciario Inferior, Grupo Salta). Ameghiniana. 1978; 15(3-4): 285-300.

Quattrocchio M, del Papa CE. Paleoambiente de la Secuencia Maíz Gordo (¿ Paleoceno Tardío-Eoceno Temprano?), Arroyo Las Tortugas, Cuenca del Grupo Salta (NO Argentina). Palinología y sedimentología. Spanish Journal of Palaeontology. 2000; 15(1): 57-70.

Quattrocchio ME, Volkheimer W. Microflora de los estratos limítrofes entre Cretácico y Terciario en las localidades de Tilián y Corralito, cuenca del Grupo Salta. Descripciones Sistemáticas. In: Volkheimer W, Sepulveda E, Archangelsky S, Cuneo N, Beresi M, Heredia S, et al., editors. 4º Congreso Argentino de Paleontología y Bioestratigrafia; 1986 Nov 23-27; Mendoza, Argentina. Buenos Aires: Asociación Paleontológica Argentina; 1988. p. 109-120.

Quattrocchio ME, Volkheimer W. Paleogene paleoenvironmental trends as reflected by palynological assemblage types, Salta Basin, NW Argentina. Neues Jahrb Geol Palaontol Abh. 1990: 377-396.

Quattrocchio ME, Marquillas R, Volkheimer W. Palinología, paleoambientes y edad de la Formación Tunal, Cuenca del Grupo Salta (Cretácico-Eoceno) República Argentina. In: Volkheimer W, Sepulveda E, Archangelsky S, Cuneo N, Beresi M, Heredia S, et al., editors. 4º Congreso Argentino de Paleontología y Bioestratigrafia; 1986 Nov 23-27; Mendoza, Argentina. Buenos Aires: Asociación Paleontológica Argentina; 1988. p. 95-107.

Quattrocchio M, Volkheimer W, del Papa C. Palynology and paleoenvironment of the “Faja Gris”; Mealla Formation (Salta Group) at Garabatal Creek (NW Argentina). Palynology. 1997; 21(1): 231-247.

Quattrocchio ME, Volkheimer W, Marquillas RA, Salfity JA. Palynostratigraphy, palaeobiogeography and evolutionary significance of the Late Senonian and Early Palaeogene palynofloras of the Salta Group, northern Argentina. Revista Española de Micropaleontología 2005; 37(2): 259-272.

Volkheimer W, Quattrocchio M, Salfity J. Datos palinológicos de la Formación Maíz Gordo, Terciario inferior de la Cuenca de Salta. In: Ramos VA, editor. Actas del IX Congreso Geológico Argentino; 1984 Nov 5-9; San Carlos de Bariloche, Argentina. Buenos Aires: Asociación Geológica Argentina; 1984. p. 523-538.

Volkheimer W, Novara MG, Narváez PL, Marquillas RA. Palynology and paleoenvironmental significance of the Tunal Formation (Danian) at its type locality, El Chorro creek (Salta, Argentina). Ameghiniana. 2006; 43(3): 567-584.
