# Supplementary material for: A snapshot of mid Eocene landscapes in the southern Central Andes: Spore-pollen records from the Casa Grande Formation (Jujuy, Argentina)
Source: PLoS One. 2023 Apr 5;18(4):e0277389. doi: 10.1371/journal.pone.0277389 (PMC10075436; doi:10.1371/journal.pone.0277389)
Supplement: S3 Table — (DOCX) [file pone.0277389.s004.docx]

| **Unit** | **Fertile samples^a^** | **Include spore-pollen counts (Y/N) for each species** | **References** |
| --- | --- | --- | --- |
| **Lumbrera Fm.** | 6 | N | [1,2] |
|  | 6 | N | [3] |
| **Maíz Gordo Fm.** | 1 | N | [4] |
|  | 1 | N | [3] |
|  | 2 | N | [5] |
| **Mealla Fm.** | 4 | Y | [6] |
| **Tunal Fm.** | 3 | N | [7] |
|  | 3 | N | [8] |
|  | 1 | N | [3] |
|  | Non specified | N | [9] |
|  | 1 | N | [10] |
|  | 6 | N | [11] |
| **Olmedo Fm.** | 5 | N | [12] |
|  | 6 | N | [11] |

**S3 Table.** **Comparative sampling-count chart among lithostratigraphic units of the Salta Basin.**

**^a^With a significant amount of palynomorphs per sample**

**References:**

1. Quatrocchio, M. Estudio palinológico preliminar de la Formación Lumbrera (Grupo Salta), localidad Pampa Grande, Provincia de Salta, República de Argentina. In: Bertels A, Romero EJ, Baez AM, Cione A, Caccavari de Felice M, Ganduglia P, et al., editors. II Congreso Argentino de Paleontologıa y Biostratigrafıa, I Congreso Latinoamericano de Paleontologıa; 1978 Apr 2-6; Buenos Aires, Argentina. Buenos Aires: Asociación Paleontológica Argentina; 1980. p. 131-149.
2. Quattrocchio M. Contribución al conocimiento de la palinología estratigráfica de la Formación Lumbrera (Terciario Inferior, Grupo Salta). Ameghiniana. 1978; 15(3-4): 285-300.
3. Quattrocchio ME, Volkheimer W. Paleogene paleoenvironmental trends as reflected by palynological assemblage types, Salta Basin, NW Argentina. Neues Jahrb Geol Palaontol Abh. 1990: 377-396.
4. Volkheimer W, Quattrocchio M, Salfity J. Datos palinológicos de la Formación Maíz Gordo, Terciario inferior de la Cuenca de Salta. In: Ramos VA, editor. Actas del IX Congreso Geológico Argentino; 1984 Nov 5-9; San Carlos de Bariloche, Argentina. Buenos Aires: Asociación Geológica Argentina; 1984. p. 523-538.
5. Quattrocchio M, del Papa CE. Paleoambiente de la Secuencia Maíz Gordo (¿ Paleoceno Tardío-Eoceno Temprano?), Arroyo Las Tortugas, Cuenca del Grupo Salta (NO Argentina). Palinología y sedimentología. Spanish Journal of Palaeontology. 2000; 15(1): 57-70.
6. Quattrocchio M, Volkheimer W, del Papa C. Palynology and paleoenvironment of the “Faja Gris”; Mealla Formation (Salta Group) at Garabatal Creek (NW Argentina). Palynology. 1997; 21(1): 231-247.
7. Quattrocchio ME, Volkheimer W. Microflora de los estratos limítrofes entre Cretácico y Terciario en las localidades de Tilián y Corralito, cuenca del Grupo Salta. Descripciones Sistemáticas. In: Volkheimer W, Sepulveda E, Archangelsky S, Cuneo N, Beresi M, Heredia S, et al., editors. 4º Congreso Argentino de Paleontología y Bioestratigrafia; 1986 Nov 23-27; Mendoza, Argentina. Buenos Aires: Asociación Paleontológica Argentina; 1988. p. 109-120.
8. Quattrocchio ME, Marquillas R, Volkheimer W. Palinología, paleoambientes y edad de la Formación Tunal, Cuenca del Grupo Salta (Cretácico-Eoceno) República Argentina. In: Volkheimer W, Sepulveda E, Archangelsky S, Cuneo N, Beresi M, Heredia S, et al., editors. 4º Congreso Argentino de Paleontología y Bioestratigrafia; 1986 Nov 23-27; Mendoza, Argentina. Buenos Aires: Asociación Paleontológica Argentina; 1988. p. 95-107.
9. Narváez PL, Volkheimer W. Nuevos datos palinológicos de la Formación Tunal, Daniano de la Cuenca del Grupo Salta. In: de Barrio RE, Etcheverry RO, Caballé MF, Llambías EJ, editors. 16° Congreso Geológico Argentino; 2005 Sep, La Plata, Argentina. Buenos Aires: Asociación Geológica Argentina, 2005. p. 465-466.
10. Volkheimer W, Novara MG, Narváez PL, Marquillas RA. Palynology and paleoenvironmental significance of the Tunal Formation (Danian) at its type locality, El Chorro creek (Salta, Argentina). Ameghiniana. 2006; 43(3): 567-584.
11. Narváez PL. Palinoestratigrafía, paleoambientes y cambios climáticos durante el Cretácico final y Paleógeno de la Cuenca del grupo Salta, República Argentina. [Ph.D. thesis]. Mendoza: Universidad Nacional de Cuyo; 2009. Available from: <https://planificacion.bdigital.uncu.edu.ar/objetos_digitales/5502/narvaez-tesisd.pdf>.
12. Moroni AM. Mtchedlishvilia saltenia n. sp. en sedimentitas del Grupo Salta, provincia de Salta. In: Herbst R, Nullo F, Bondesio P, Alvarez BB, Anzótegui LM, Balent ST, et al., editors. Congreso Argentino de Paleontología y Bioestratigrafía; 1982 Sep 6-10, Corrientes, Argentina. Buenos Aires: Asociación Paleontológica Argentina; 1984. p. 129-139.
