# Supplementary material for: A snapshot of mid Eocene landscapes in the southern Central Andes: Spore-pollen records from the Casa Grande Formation (Jujuy, Argentina)
Source: PLoS One. 2023 Apr 5;18(4):e0277389. doi: 10.1371/journal.pone.0277389 (PMC10075436; doi:10.1371/journal.pone.0277389)
Supplement: Attachment — Submitted filename: PONE-D-22-29456-RE.pdf [file pone.0277389.s006.pdf]

# PLOS ONE

## A snapshot of mid Eocene landscapes in the southern Central Andes: spore-pollen records from Casa Grande Formation (Jujuy, Argentina)

--Manuscript Draft--

| | |
|---|---|
| **Manuscript Number:** | PONE-D-22-29456 |
| **Article Type:** | Research Article |
| **Full Title:** | A snapshot of mid Eocene landscapes in the southern Central Andes: spore-pollen records from Casa Grande Formation (Jujuy, Argentina) |
| **Short Title:** | Eocene floras from the southern Central Andes |
| **Corresponding Author:** | Luis Palazzesi<br>Consejo Nacional de Investigaciones Cientificas y Tecnicas<br>Buenos Aires, ARGENTINA |
| **Keywords:** | Puna; South America; Eocene; fossils; palynomorphs; Andes; floras; landscapes |
| **Abstract:** | The southern Central Andes –or Puna– now contains specialized plant communities adapted to life in extreme environments. During the mid Eocene (~40 Ma), the Cordillera at these latitudes was barely uplifted and global climates were much warmer than today. No fossil plant remains have been discovered so far from this age in the Puna region to attest to past scenarios. Yet, we assume that the vegetation cover must have been very different from what it looks today. To test this hypothesis, we study a spore-pollen record from the mid Eocene Casa Grande Formation (Jujuy, northwestern Argentina). Although sampling is preliminary, we found ~70 morphotypes of spores, pollen grains and other palynomorphs, many of which were produced by taxa with tropical or subtropical modern distributions (e.g. Arecaceae, Ulmaceae Phyllostylon, Malvaceae Bombacoideae). Our reconstructed scenario implies the existence of a vegetated pond surrounded by trees, vines, and palms. We also report the northernmost records of a few unequivocal Gondwanan taxa (e.g. Nothofagus, Microcachrys), about 5,000 km north from their Patagonian-Antarctic hotspot. With few exceptions, the discovered taxa –both neotropical and gondwanan– became extinct from the region following the severe effects of the Andean uplift and the climate deterioration during the Neogene. We found no evidence for enhanced aridity nor cool conditions in the southern Central Andes at mid Eocene times. Instead, the overall assemblage represents a frost-free and humid to seasonally-dry ecosystem that prevailed near a lacustrine environment, in agreement with previous paleoenvironmental studies. Our reconstruction adds a further biotic component to the previously reported record of mammals. |
| **Order of Authors:** | Ezequiel E. Farrell |
| | Mariano Tapia, Biologist |
| | Lilia Mautino |
| | Cecilia del Papa |
| | Viviana D. Barreda |
| | Luis Palazzesi |
| **Additional Information:** | |
| **Question** | **Response** |
| **Financial Disclosure**<br><br>Enter a financial disclosure statement that describes the sources of funding for the work included in this submission. Review the submission guidelines for detailed requirements. View published research | The authors received no specific funding for this work. |

Powered by Editorial Manager® and ProduXion Manager® from Aries Systems Corporation

| | |
|---|---|
| articles from *PLOS ONE* for specific examples.<br><br>This statement is required for submission and **will appear in the published article** if the submission is accepted. Please make sure it is accurate.<br><br>**Unfunded studies**<br>Enter: *The author(s) received no specific funding for this work.*<br><br>**Funded studies**<br>Enter a statement with the following details:<br>• Initials of the authors who received each award<br>• Grant numbers awarded to each author<br>• The full name of each funder<br>• URL of each funder website<br>• Did the sponsors or funders play any role in the study design, data collection and analysis, decision to publish, or preparation of the manuscript?<br>• **NO** - Include this sentence at the end of your statement: *The funders had no role in study design, data collection and analysis, decision to publish, or preparation of the manuscript.*<br>• **YES** - Specify the role(s) played.<br><br>* typeset | |
| **Competing Interests**<br><br>Use the instructions below to enter a competing interest statement for this submission. On behalf of all authors, disclose any competing interests that could be perceived to bias this work—acknowledging all financial support and any other relevant financial or non-financial competing interests.<br><br>This statement is required for submission and **will appear in the published article** if the submission is accepted. Please make sure it is accurate and that any funding sources listed in your Funding Information later in the submission form are also declared in your Financial Disclosure statement. | The authors have declared that no competing interests exist. |

| | |
|---|---|
| View published research articles from *PLOS ONE* for specific examples.<br><br>**NO authors have competing interests**<br><br>Enter: *The authors have declared that no competing interests exist.*<br><br>**Authors with competing interests**<br><br>Enter competing interest details beginning with this statement:<br><br>*I have read the journal's policy and the authors of this manuscript have the following competing interests: [insert competing interests here]*<br><br>* typeset | |
| **Ethics Statement**<br><br>Enter an ethics statement for this submission. This statement is required if the study involved:<br><br>• Human participants<br>• Human specimens or tissue<br>• Vertebrate animals or cephalopods<br>• Vertebrate embryos or tissues<br>• Field research<br><br>Write "N/A" if the submission does not require an ethics statement.<br><br>General guidance is provided below. Consult the submission guidelines for detailed instructions. **Make sure that all information entered here is included in the Methods section of the manuscript.** | N/A |

Powered by Editorial Manager® and ProduXion Manager® from Aries Systems Corporation

| | |
|---|---|
| **Format for specific study types**<br><br>**Human Subject Research (involving human participants and/or tissue)**<br>• Give the name of the institutional review board or ethics committee that approved the study<br>• Include the approval number and/or a statement indicating approval of this research<br>• Indicate the form of consent obtained (written/oral) or the reason that consent was not obtained (e.g. the data were analyzed anonymously)<br><br>**Animal Research (involving vertebrate animals, embryos or tissues)**<br>• Provide the name of the Institutional Animal Care and Use Committee (IACUC) or other relevant ethics board that reviewed the study protocol, and indicate whether they approved this research or granted a formal waiver of ethical approval<br>• Include an approval number if one was obtained<br>• If the study involved *non-human primates*, add *additional details* about animal welfare and steps taken to ameliorate suffering<br>• If anesthesia, euthanasia, or any kind of animal sacrifice is part of the study, include briefly which substances and/or methods were applied<br><br>**Field Research**<br><br>Include the following details if this study involves the collection of plant, animal, or other materials from a natural setting:<br>• Field permit number<br>• Name of the institution or relevant body that granted permission | |
| **Data Availability**<br><br>Authors are required to make all data underlying the findings described fully available, without restriction, and from the time of publication. PLOS allows rare exceptions to address legal and ethical concerns. See the PLOS Data Policy and FAQ for detailed information. | Yes - all data are fully available without restriction |

| | |
|---|---|
| A Data Availability Statement describing where the data can be found is required at submission. Your answers to this question constitute the Data Availability Statement and **will be published in the article**, if accepted.<br><br>**Important:** Stating 'data available on request from the author' is not sufficient. If your data are only available upon request, select 'No' for the first question and explain your exceptional situation in the text box.<br><br>Do the authors confirm that all data underlying the findings described in their manuscript are fully available without restriction? | |
| **Describe where the data may be found in full sentences. If you are copying our sample text, replace any instances of XXX with the appropriate details.**<br><br>• If the data are **held or will be held in a public repository**, include URLs, accession numbers or DOIs. If this information will only be available after acceptance, indicate this by ticking the box below. For example: *All XXX files are available from the XXX database (accession number(s) XXX, XXX.).*<br>• If the data are all contained **within the manuscript and/or Supporting Information files**, enter the following: *All relevant data are within the manuscript and its Supporting Information files.*<br>• If neither of these applies but you are able to provide **details of access elsewhere**, with or without limitations, please do so. For example:<br><br>*Data cannot be shared publicly because of [XXX]. Data are available from the XXX Institutional Data Access / Ethics Committee (contact via XXX) for researchers who meet the criteria for access to confidential data.*<br><br>*The data underlying the results presented in the study are available from (include the name of the third party* | The fossil specimens analysed and illustrated in the present study will be held in a public repository under the code PAL JUA (P) Cz/Plg. N° 001a-g(Eo). |

| | |
|---|---|
| *and contact information or URL).*<br>• This text is appropriate if the data are owned by a third party and authors do not have permission to share the data.<br><br>* typeset | |
| Additional data availability information: | Tick here if the URLs/accession numbers/DOIs will be available only after acceptance of the manuscript for publication so that we can ensure their inclusion before publication. |

Manuscript
Click here to access/download;Manuscript;Farrell_et_al_2022.docx

# A snapshot of mid Eocene landscapes in the southern Central Andes: spore-pollen records from Casa Grande Formation (Jujuy, Argentina)

Ezequiel Farrell[1¶], Mariano J. Tapia[2¶], Lilia R. Mautino[1], Cecilia del Papa[3], Viviana D. Barreda[2], Luis Palazzesi[2*]

*Corresponding author

E-mail: lpalazzesi@macn.gov.ar (LP).

[¶]These authors contributed equally to this work.

[1]Centro de Ecología Aplicada del Litoral, Corrientes, Argentina

[2]Department of Paleobotany, Museo Argentino de Ciencias Naturales, Buenos Aires, Argentina

[3]Centro de Investigaciones en Ciencias de la Tierra, Córdoba, Argentina

**Short title**: Eocene floras from the southern Central Andes

# Abstract

The southern Central Andes –or Puna– now contains specialized plant communities adapted to life in extreme environments. During the mid Eocene (~40 Ma), the Cordillera at these latitudes was barely uplifted and global climates were much warmer than today. No fossil plant remains have been discovered so far from this age in the Puna region to attest to past scenarios. Yet, we assume that the vegetation cover must have been very different from what it looks today. To test this hypothesis, we study a spore-pollen record from the mid Eocene Casa Grande Formation (Jujuy, northwestern Argentina). Although sampling is preliminary, we found ~70 morphotypes of spores, pollen grains and other palynomorphs, many of which were produced by taxa with tropical or subtropical modern distributions (e.g. Arecaceae, Ulmaceae *Phyllostylon*, Malvaceae Bombacoideae). Our reconstructed scenario implies the existence of a vegetated pond surrounded by trees, vines, and palms. We also report the northernmost records of a few unequivocal Gondwanan taxa (e.g. *Nothofagus*, *Microcachrys*), about 5,000 km north from their Patagonian-Antarctic hotspot. With few exceptions, the discovered taxa –both neotropical and gondwanan– became extinct from the region following the severe effects of the Andean uplift and the climate deterioration during the Neogene. We found no evidence for enhanced aridity nor cool conditions in the southern Central Andes at mid Eocene times. Instead, the overall assemblage represents a frost- free and humid to seasonally-dry ecosystem that prevailed near a lacustrine environment, in agreement with previous paleoenvironmental studies. Our reconstruction adds a further biotic component to the previously reported record of mammals.

# Introduction

The Puna-Altiplano encompasses a high elevation montane grassland in the Central Andes, extending from southern Peru, through Bolivia into northern Chile and Argentina [1,2]. The landscape is a high plateau ranging from 3,700 m to 4,500 m above sea level, with snow-covered peaks and saline lakes. The climate ranges from temperate to cold and arid; average temperature ranges between 0ºC and 15ºC while precipitation ranges between 200 and 300 mm per year [3]. These climatic conditions associated with important disturbances like droughts and frosts have restricted the development of life but have favored the establishment of species able to cope with these conditions. For example, the vegetation is characterized by tall tussocks of bunchgrass, and shrubs such as *Parastrephia lepydophilla* (Asteraceae) as well as other cushion plants and sparse lichens and mosses [4]. Fauna, on the other hand, include vicuña (*Lama vicugna*), guanaco (*Lama guanicoe*), chinchilla (*Chinchilla chinchilla*), vizcacha (*Lagidium viscacia*), the andean cat (*Felis jacobita*) and the large flightless bird Darwin's rhea (*Rhea pennata*), among others [5-7].

The harsh climatic conditions prevailing in the Puna are mostly attributed to the Neogene uplift of the Andean Cordillera, which blocks the easterly humid winds, casting a shadow of dryness on the leeward [8-10]. However, the reconstructed climates from the Paleogene are remarkably different from those of today; during the Paleocene and early Eocene, sedimentological information (e.g., clay minerals and paleosols) from the Salta Group, in Northwestern Argentina, indicate humid-subtropical to tropical climates, although episodes of seasonality have been identified [11-14]. This paleoclimate supported a diverse biota with fish, crocodiles, lizards and mammals [15]. Also, spores and pollen grains demonstrated the existence of landscapes dominated by humid forests and lacustrine environments, with local arid conditions [16-21]. The onset of the Andean Cordillera uplift in the mid Eocene at these latitudes (22°S–26°S) [22-23] have driven important environmental (e.g., [24]), and climatic shifts [25], although little is known about its impact on biotas (e.g. [26]). Here, we present terrestrially-derived palynomorphs (largely pollen and spores) from

deposits of the Casa Grande Formation (Fig 1), mid Eocene (approx. 40 Myr), in northwestern Argentina. The available sedimentological information points to the existence of a depositional environment characterized by plains with localized ponds or shallow lakes and meandering fluvial systems with clay-rich floodplains [27]. Large herbivores as browsers (Leontiniidae) and some scavengers (Dasypodidae) along with small grazers, fruit and insect feeders (Prepidolopidae) have occurred across these landscapes [15,28,29]. Yet, little is known about the composition and structure of the plant cover, which is the primary support of habitats where these and other herbivores may have lived, fed on, and found shelter.

**Fig 1. Geographic and stratigraphic distribution of the Casa Grande Fm.** (A) Location map (Northwestern Argentina). (B) Study area in the Jujuy Province, showing the geographic distribution of the Casa Grande Fm. and the sampled site. (C) Stratigraphic chart of the Groups/Formations recognized in the region.

## Geological and chronostratigraphic context

The Casa Grande Formation [30] includes red to reddish brown siltstones and sandstones (Fig 2) with a mean thickness of 800 m [31], and cropps out along the Tres Cruces–Mina Aguilar area, in the Jujuy province, northeastern Argentina (Fig 1). This unit represents the onset deposition in the foreland basin [27,31] contemporaneous with the initial construction of the Andean topography [23). Montero-López et al. [27] proposed a subdivision of this sedimentary unit into the Casa Grande 1 and Casa Grande 2 sequences on the basis of a new and more detailed mapping analysis. The Casa Grande 1 sequence was previously assigned to the Lumbrera Formation of the Salta Group (e.g., [31,32]). However, Montero-López et al. [27] recognized a sharp stratigraphic contact between the underlying dark reddish brown, highly cemented mudstones of the early Eocene Lumbrera Formation and the overlying reddish, gypsum-rich sandy siltstones of the Casa Grande 1 sequence. The ~160 m- thick Casa Grande 1 sequence, which preserves the palynomorphs we present and

illustrate in this contribution, includes dark reddish orange fine-grained sediments, interbedded with green to dark gray claystones, carbonate and fine-grained sandstones (Fig 3). The depositional environment for this sequence is interpreted to be transitional between a vegetated mudflat with ponds and shallow lakes, and isolated channel belts. Until now, no radiometric ages have been published for the Casa Grande 1 sequence, although absolute age for the upper Lumbrera Formation –equivalent unit–- provided an absolute U/Pb zircon depositional age of 39.9 ± 0.4 Ma (Eocene) [33]. Furthermore, fossil mammalian remains discovered from the Casa Grande 1 also support the Eocene age (e.g., [28],[34]).

**Fig 2. Field aspect of the palynomorph-bearing sediments.** Note the overall appearance of the terrestrial deposits cropping out in Jujuy (Casa Grande Fm.).

**Fig 3. Stratigraphic and sedimentological characteristics of the Casa Grande Formation.** Note the location of the palynologically-rich level located in the upper part of the section.

# Material and Methods

Palynological samples were collected from seven stratigraphic levels of the Casa Grande 1 sequence from a 165 m-thick section of red to reddish gypsum-rich sandy siltstones and green-gray mudstones, exposed in the Casa Grande valley (Figs 1-3). Palynomorphs are best preserved in unoxidized, fine-grained, primarily dark-colored (gray to black) sediments; the Casa Grande 1 sequence lacks suitable lithologies to preserve organic material, and hence any productive palynological level is very valuable to reconstruct past floras and environments. Seven samples were processed and sieved using standard palynological techniques [35-36]), of which only one, from a lacustrine bed, yielded palynomorphs; the residue was permanently mounted in six palynological replicates using UV-curable acrylates gel [37]. The slides are housed in the palynological collection of Jujuy under the code PAL JUA (P) Cz/Plg. N° 001a-g(Eo). Coordinates of the illustrated specimens are referred to a

Leica DM500 microscope. Pollen terminology follows Punt et al. [38]. Fossil spores and pollen grains were identified to species level and diagnosed to modern families, subfamilies, tribes or genera whenever possible (Table 1). We used R software program, version 2.2.0 [39] for estimating similarities among the spore-pollen assemblages from the Casa Grande 1 sequence and those previously reported from other Paleogene localities cropping out in northwestern Argentina [i.e., Tunal/Olmedo Formation (early Paleocene [19-21]); Mealla Formation (late Paleocene [17]); Maíz Gordo Formation (Paleocene/Eocene [18]); Lumbrera Formation (Early Eocene [16,17])] based on presence/absence data (S1 Table). The Hierarchical Clustering ('hclust' function) was run using a distance matrix ('dist' function, 'method: binary) from the R package 'stats' version 4.0.2.

**Table 1. Identified species list.**

| **Fossil taxon** | **Botanical affinity (Nearest Living Relative)** |
|---|---|
| **Trilete spores** | |
| *Deltoidospora minor* (Couper) Pocock 1970 (Fig 4B) | Cyatheaceae, Dicksoniaceae, Schizaeaceae |
| *Leiotriletes* sp. in Mautino 2009 (Fig 4C) | Pteridophyta |
| *Polypodiaceoisporites* cf. *retirugatus* Muller 1968 (Fig 4D) | Pteridaceae (*Pteris* spp.) |
| *Reboulisporites fuegiensis* Zamaloa and Romero 1990 (Fig 4E) | Ricciaceae |
| *Todisporites minor* Couper 1958 (Fig 4A) | Osmundaceae |
| *Zlivisporis* sp. (Fig 4F) | Bryophyta. Hepaticae |
| **Sacate pollen** | |
| *Microcachryidites antarcticus* Cookson 1947 (Fig 4H) | Podocarpaceae (*Microcachrys tetragona*) |
| *Podocarpidites* sp. (Fig 4G) | Podocarpaceae |
| **Plicate pollen** | |
| *Equisetosporites notensis* (Cookson) Romero 1977 (Fig 4I) | Ephedraceae |
| **Inaperturate pollen** | |

| | |
|---|---|
| *Smilacipites* sp. cf. *S. herbaceoides* Wodehouse 1933 (Fig 4J) | Smilacaceae, *Smilax, Peltandra* |
| **Monosulcate pollen** | |
| *Arecipites minutiscabratus* (McIntyre 1968) Milne 1988 (Fig 4K) | Arecaceae |
| *Echimonocolpites* sp. (Fig 4N) | Arecaceae/unknown angiosperm? |
| *Liliciditesmirus* Srivastava 1969 (Fig 4M) | Liliaceae |
| *Liliacidites vermireticulatus* Archangelsky and Zamaloa 1986 (Fig 4L) | Iridaceae |
| *Punctilongisulcites punctiechinatus* (Krutzsch) Casas Gallego and Barrón 2020 | Hydrocharitaceae |
| *Verrumonocolpites* sp. | Unknown |
| **Colpate pollen** | |
| *Beaupreaidites sp.* (Fig 5A) | Proteaceae (*Beauprea*) |
| *Nothofagidites anisoechinatus* Menendez and Caccavari 1965 (Fig 5O) | Nothofaceae Brassi Type |
| *Nothofagidites saraensis* Menendez and Caccavari 1975 (Fig 5P) | Nothofagaceae Fusca Type |
| *Retitrescolpites adultus* Gonzalez Guzman 1967 (Fig 5Q) | Unknown |
| *Tricolpites aspermarginis* McIntyre 1968 (Fig 5R) | Violaceae |
| *Tricolpites membranus* Couper 1960 (Fig 5S) | Phytollacaceae (*Seguieria*) |
| *Tricolpites* sp. in Mautino 2009 (Fig 5T) | Oxalidaceae, *Oxalis* spp. |
| **Colporate pollen** | |
| *Ailanthipites* sp. (Fig 5E) | Anacardiaceae |
| *Baumannipollis* sp. (Fig 5J) | Malvaceae (*Lagunaria*, *Modiolastrum, Urocarpidinium, Tarasa*) |
| *Bombacacidites* sp. (Fig 5F) | Malvaceae Bombacoideae |
| *Foveotricolporites* sp. (Fig 5C) | Unknown |
| *Heterocolpites rotundus* Hoorn 1993 (Fig 5I) | Combretaceae-Melastomataceae |
| *Malvacipolloides tucumanensis* Mautino et al. 2004 | Malvaceae, Malveae |
| *Margocolporites tenuireticulatus* Barreda 1997 (Fig 5D) | Fabaceae Mimosoideae |
| *Rhoipites baculatus* Archangelsky 1973 (Fig 5G) | Fabaceae Papilionoideae (*Aeschynomene*) |

| | |
|---|---|
| *Rhoipites guianensis* (Van der Hammen and Wymstra) Jaramillo and Dilcher 2001 (Fig 5H) | Malvaceae Sterculioideae (*Firmiana* and *Hildegardia*) |
| *Siltaria dilcheri* Silva-Caminha et al. 2010 (Fig 5B) | Unknown Dicotyledonae |
| **Porate pollen** | |
| *Corsinipollenites menendezii* Quattrocchio 1978 (Fig 5M) | Onagraceae (*Ludwigia*) |
| *Gomphrenipollis* sp. 1 (Fig 5S) | Amaranthaceae (*Gomphrena*) |
| *Gomphrenipollis* sp. 2 (Fig 5T) | Amaranthaceae (*Gomphrena*) |
| *Graminidites* sp. (Fig 5K) | Poaceae |
| *Pandaniidites* sp. (Fig 5L) | Araceae (*Lemna*) |
| *Periporopollenites polyoratus* (Couper 1960) Stover in Stover and Partridge 1973 (Fig 5R) | Caryophyllaceae-Trimeniaceae |
| *Periporopollenites* sp. (Fig 5Q) | Caryophyllaceae (*Silene*) |
| *Psilaperiporites circinatus* D'Apolito et al. 2021 (Fig 5P) | Unknown |
| *Psilatriporites desilvae* Hoorn 1993 (Fig 5N) | Fabaceae Caesalpinioideae |
| *Verrustephanosporites simplex* Leidermeyer 1966 (Fig 5O) | Ulmaceae (*Phyllostylon*) |
| **Fungal remains** | |
| *Biporipsilonites krempii* (Varma and Rawat 1963) Kalgutkar and Jansonius 2000 (Fig 6A) | Fungi |
| *Colligerites kutchensis* (Kar and Saxena) Jain and Kar 1979 | Fungi. |
| *Dictyosporites* sp. Kalgutkar and Braman 2008 | Fungi |
| *Inapertisporites edigeri* Kalgutkar and Jansonius 2000 (Fig 6B) | Fungi |
| *Inapertisporites subovoideus* (Sheffy and Dilcher) Kalgutkar and Jasonius 2000 | Fungi |
| *Multicellites crassisporus* (Salard-Cheboldaeff and Locquin) Kalgutkar and Jansonius 2000 (Fig 6C) | Fungi |
| *Pluricellaesporites sheffyi* Martínez Hernández and Tomasini Ortiz 1989 | Fungi |
| *Polycellaesporonites bellus* Chandra et al. 1984 (Fig 6E) | Fungi |
| *Quilonia* sp. cf. *Q. allepeyensis* (Ramanujam and Rao) Kalgutkar and Jasonius 2000 (Fig 6D) | Fungi |
| *Reduviasporonites* sp. cf. *R. catenulatus* Wilson 1962 | Fungi |

| | |
|---|---|
| *Scolecosporites modicus* Kalgutkar and Jansonius 2000 (Fig 6F) | Fungi |
| **Zooclasts** | |
| *Filinia* resting eggs (Fig 6K) | Rotifera, Trochosphaeridae |
| *Hydrozetes*, adult leg (Fig 6J) | Acari, Oribatida |
| Scales of Lepidopteran wings (Figs 6G,H) | Lepidoptera |
| Scolecodont-like palynomorphs (Plate 3, Fig 6I) | Arthropoda? |
| **Other terrestrial palynomorphs** | |
| Compact type sclerocyte (Fig 6L) | - |
| **Fresh-water algae** | |
| *Cymatiosphaera sp*. Mays et al. 2021 | Cymatiospharaceae |
| *Mougeotia* sp. (Fig 6N) | Zygnemataceae |
| *Pediastrum biradiatum* Meyen 1829 | Hydrodictyaceae |
| *Pediastrum boryanum* (Turp.) Menegh 1840 (Fig 6M) | Hydrodictyaceae |
| *Pediastrum duplex* var. *gracilimum* West and West 1895 | Hydrodictyaceae |
| *Planctonites stellarius* (Potonié 1934) Krutzsch 1960 (Fig 6O) | Zygnematales, Desmidiaceae. |
| *Scenedesmus* sp. (Fig 6R) | Scenedesmaceae |
| *Spirogyra* sp. 6 Martinez et al. 2008 (Fig 6Q) | Zygnemataceae |
| *Stigmozygodites ministigmosus* Krutzsch and Pacltová 1990 (Fig 6P) | Zygnemataceae (*Zygnema*) |
| **Marine algae** | |
| Indeterminate acritarch | Unknwon |
| Indeterminate dinoflagellate cyst 1 (Fig 6S) | Dinoflagellata |
| Indeterminate dinoflagellate cyst 2 (Fig 6T) | Dinoflagellata |

Palynomorphs recovered from the Casa Grande Formation along with their botanical affinities (Nearest Living Relative).

# Results and discussion

The palynological assemblage recovered from Casa Grande 1 sequence is well preserved and relatively diverse. It includes spores, pollen grains, algae, among other fossil remains (fungi and zoological remains). We identified 43 spore and pollen species (Table 1, Figs 4-6), ~~broken down as~~ 2 bryophytes, 4 pteridophytes, 3 gymnosperms, and 34 angiosperms. From these, we found the nearest living relatives (families, tribes or genera) of 37 species (2 ferns, 2 bryophytes, 3 gymnosperms, and 30 angiosperms); the remaining sporomorphs have doubtful or uncertain botanical affinity. From the 43 recognized taxa, 38 has been reported for the first time at Casa Grande 1 Fm., compared to older Paleogene sediments cropping out nearby (i.e., Olmedo, Tunal, Mealla, Maíz Gordo, Lumbrera). These records, combined with those recorded earlier in northwestern Argentina, provide a total of 55 confidently assigned plant families for most of the early Paleogene (Paleocene-mid Eocene).

**Fig 4. Microscope images of selected spores and pollen grains from the Casa Grande Formation.** (A) *Todisporites minor* (PAL JUA (P) Cz/Plg. N° 001c(Eo)+10 µm: 21.9-150.9). (B) *Deltoidospora minor* (PAL JUA (P) Cz/Plg. N° 001d(Eo)+10 µm: 10-135). (C) *Leiotriletes* sp. (PAL JUA (P) Cz/Plg. N° 001c(Eo)+10 µm: 9-138.9). (D) *Polypodiaceoisporites* cf. *P. retirugatus* (PAL JUA (P) Cz/Plg. N° 001b(Eo)+10 µm: 21.5-131). (E) *Reboulisporites fuegiensis* (PAL JUA (P) Cz/Plg. N° 001e(Eo)+25 µm: 3.5-139.4). (F) *Zlivisporis* sp. (PAL JUA (P) Cz/Plg. N° 001f(Eo)+25 µm: 16-151). (G) *Podocarpidites* sp. (PAL JUA (P) Cz/Plg. N° 001b(Eo)+10 µm: 3-153.5). (H) *Microcachryidites antarcticus* (PAL JUA (P) Cz/Plg. N° 001d(Eo)+10 µm: 3.9-130.5). (I) *Equisetosporites notensis* (PAL JUA (P) Cz/Plg. N° 001e(Eo)+10 µm: 4.5-154.6). (J) *Smilacipites* sp. cf. *S. herbaceoides* (PAL JUA (P) Cz/Plg. N° 001d(Eo)+10 µm: 16.9-135.5). (K) *Arecipites minutiscabratus* (PAL JUA (P) Cz/Plg. N° 001d(Eo)+10 µm: 23-152.2). (L) *Liliacidites vermireticulatus* (PAL JUA (P) Cz/Plg. N° 001d(Eo)+10 µm: 24.2-126.9). (M) *Liliciditess mirus* (PAL JUA (P) Cz/Plg. N° 001d(Eo)+25 µm: 15-139). (N) *Echimonocolpites* sp. (PAL JUA (P) Cz/Plg. N° 001b(Eo)+10 µm: 5.2-128.9). (O) *Nothofagidites anisoechinatus* (PAL JUA (P) Cz/Plg. N° 001e(Eo)+10 µm: 13-

128). (P) *Nothofagidites saraensis* (PAL JUA (P) Cz/Plg. N° 001e(Eo)+10 µm: 15.5-152.6). (Q) *Retitrescolpites adultus* (PAL JUA (P) Cz/Plg. N° 001d(Eo)+25 µm: 14-150.4). (R)*Tricolpites asperamarginis* (PAL JUA (P) Cz/Plg. N° 001d(Eo)+10 µm: 13.8-128.8). (S) *Tricolpites membranus* (PAL JUA (P) Cz/Plg. N° 001f(Eo)+10 µm: 14.9-152). (T) *Tricolpites* sp. (PAL JUA (P) Cz/Plg. N° 001c(Eo)+10 µm: 17-143. Scale bars: 10 µm (except in A, I, J, and N: 5 µm). Each species name is followed by slide number and coordinates of Leica DM500 microscope held at Museo Argentino de Ciencias Naturales, Buenos Aires, Argentina.

**Fig 5. Microscope images of selected pollen grains from the Casa Grande Formation.** (A) *Beaupreaidites* sp. (PAL JUA (P) Cz/Plg. N° 001b(Eo)+10 µm: 7.5-142). (B) *Siltaria dilcheri* (PAL JUA (P) Cz/Plg. N° 001d(Eo)+10 µm: 17-151.2). (C) *Foveotricolporites* sp. (PAL JUA (P) Cz/Plg. N° 001b(Eo)+10 µm: 15.5-141.5). (D) *Margocolporites tenuireticulatus* (PAL JUA (P) Cz/Plg. N° 001a(Eo)+10 µm: 23-141). (E) *Ailanthipites* sp. (PAL JUA (P) Cz/Plg. N° 001b(Eo)+10 µm: 19-149). (F) *Bombacacidites* sp. (PAL JUA (P) Cz/Plg. N° 001e(Eo)+10 µm: 8-153.2). (G) *Rhoipites baculatus* (PAL JUA (P) Cz/Plg. N° 001c(Eo)+10 µm: 24-149.9). (H) *Rhoipites guianensis* (PAL JUA (P) Cz/Plg. N° 001e(Eo)+10 µm: 10-152). (I) *Heterocolpites rotundus* (PAL JUA (P) Cz/Plg. N° 001e(Eo)+10 µm: 9.5-152.5). (J) *Baumannipollis* sp. (PAL JUA (P) Cz/Plg. N° 001c(Eo)+25 µm: 21.3-138). (K) *Graminidites* sp. (PAL JUA (P) Cz/Plg. N° 001c(Eo)+10 µm: 15-145.9). (L) *Pandaniidites* sp. (PAL JUA (P) Cz/Plg. N° 001d(Eo)+10 µm: 13.9-130.2). (M) *Corsinipollenites menendezii* (PAL JUA (P) Cz/Plg. N° 001d(Eo)+25 µm: 2.5-137). (N) *Psilatriporites desilvae* (PAL JUA (P) Cz/Plg. N° 001d(Eo)+10 µm: 16.7-155). (O) *Verrustephanosporites simplex* (PAL JUA (P) Cz/Plg. N° 001d(Eo)+10 µm: 14,8-142,7). (P) *Psilaperiporites circinatus* (PAL JUA (P) Cz/Plg. N° 001f(Eo)+10 µm: 10.1-132.2). (Q) *Periporopollenites*? sp. 1 (PAL JUA (P) Cz/Plg. N° 001e(Eo)+10 µm: 7-151). (R) *Periporopollenites polyoratus* (PAL JUA (P) Cz/Plg. N° 001e(Eo)+10 µm: 9.5-152). (S) *Gomphrenipollis* sp. 1 (PAL JUA (P) Cz/Plg. N° 001e(Eo)+10 µm: 7-141). *Gomphrenipollis* sp. 2 (PAL JUA (P) Cz/Plg. N° 001b(Eo)+10 µm: 4-130.5). Scale bars: 10 µm (except in L, N and R: 5 µm). Each species name is followed by slide

number and coordinates of Leica DM500 microscope held at Museo Argentino de Ciencias Naturales, Buenos Aires, Argentina.

**Fig 6. Microscope images of selected non-pollen palynomorphs from Casa Grande Formation.** (A) *Biporipsilonites krempii* (PAL JUA (P) Cz/Plg. N° 001b(Eo)+10 μm: 2-142.5). (B) *Inapertisporites edigeri* (PAL JUA (P) Cz/Plg. N° 001b(Eo)+10 μm: 6-145.5). (C) *Multicellites crassisporus* (PAL JUA (P) Cz/Plg. N° 001d(Eo)+10 μm: 20.8-129.2). (D) *Quilonia* sp. cf. *Q. allepeyensi* (PAL JUA (P) Cz/Plg. N° 001b(Eo)+10 μm: 4.1-126.7). (E) *Polycellaesporonites bellus* (PAL JUA (P) Cz/Plg. N° 001e(Eo)+10 μm: 7-141.5). (F) *Scolecosporites modicus* (PAL JUA (P) Cz/Plg. N° 001b(Eo)+10 μm: 7.5-153.4). (G) Scales of Lepidopteran wings (PAL JUA (P) Cz/Plg. N° 001e(Eo)+25 μm: 11-143.9). (H) Scales of Lepidopteran wings (PAL JUA (P) Cz/Plg. N° 001d(Eo)+25 μm: 19.8-141.5). (I) Scolecodonts (PAL JUA (P) Cz/Plg. N° 001e(Eo)+25 μm: 2-153.9). (J) *Hydrozetes*, leg (PAL JUA (P) Cz/Plg. N° 001d(Eo)+25 μm: 16.5-144.5). (K) *Filinia* resting eggs (PAL JUA (P) Cz/Plg. N° 001d(Eo)+25 μm: 23.2-155). (L) Compact type sclerocyte (PAL JUA (P) Cz/Plg. N° 001d(Eo)+25 μm: 22.5-152). (M) *Pediastrum boryanum* (PAL JUA (P) Cz/Plg. N° 001d(Eo)+25 μm: 4-149.5). (N) *Mougeotia* sp. (PAL JUA (P) Cz/Plg. N° 001a(Eo)+10 μm: 6-145). (O) *Planctonites stellarius* (PAL JUA (P) Cz/Plg. N° 001d(Eo)+10 μm: 18-145). (P) *Stigmozygodites ministigmosus* (PAL JUA (P) Cz/Plg. N° 001d(Eo)+25 μm: 8.8-138). (Q) *Spirogyra* sp. 6 Martínez et al. 2008 (PAL JUA (P) Cz/Plg. N° 001d(Eo)+10 μm: 23.9-134.9). (R) *Scenedesmus* sp. (PAL JUA (P) Cz/Plg. N° 001e(Eo)+25 μm: 3.5-147.5). (S) Dinoflagellate cyst 1 (PAL JUA (P) Cz/Plg. N° 001d(Eo)+25 μm: 21.7-141.8). (T) Dinoflagellate cyst 2 (PAL JUA (P) Cz/Plg. N° 001c(Eo)+25 μm: 15-146.6). Scale bars: 10 μm (except A and H: 5 μm and 25 μm, respectively).

The fossil palynomorphs preserved in the terrestrial sediments of the Casa Grande 1 formation come from a single sedimentary layer, yet they were transported from local to more distant sources by biotic (e.g., insects) or abiotic (e.g., wind, rivers) means. For example, we estimate that some of the non-pollen palynomorphs were preserved *in situ* or

very close to the fossil site because they exhibit few adaptations to travel long distances. This is the case of palynomorphs assigned to algae such as Zygnemataceae (*Mougeotia* sp., Fig 6N; *Spirogyra* sp. 6, Fig 6Q; *Stigmozygodites ministigmosus*, Fig 6P), Scenedesmaceae (*Scenedesmus* sp., Fig 6R) and Hydrodictyaceae (*Pediastrum birradiatum*; *P. boryanum*, Fig 6M and *P. duplex* var. *gracillimum*), that strictly occur in freshwater ecosystems, as well the presence of fungal spores of *Reduviasporonites* suggests shallow aquatic habitats [40]. In addition, Filinia-type resting eggs (Fig 6K) are common in fresh-brackish ponds and lakes [41,42]. Moreover, oribatid mites as *Hydrozetes* remains (Fig 6J) could be restricted to freshwater habitats too (i.e., pools, ponds, raised bogs, lakes, slowly flowing waters, submerse vegetation) [43]. Long distance transport of these palynomorphs (as coenobia or zygospores) has rarely been reported (e.g., [44,45]). Also the several fossil fungal spores recovered (e.g., *Biporipsilonites krempii*, Fig 6A; *Inapertisporites edigeri*, Fig. 6B; *Pluricellaesporites sheffyi* (not illustrated); *Multicellites crassisporus*, Fig 6C; *Scolecosporites modicus*, Fig 6F; *Polycellaesporonites bellus*, Fig 6E) may have been produced at a short distance from the place where sporulation took place [41], hence indicating local occurrence. The presence of these sporomorphs at the fossil site, particularly those assigned to algae, indicate the occurrence of a shallow lake, or a pond, in agreement with previous sedimentological studies at the Casa Grande Formation [27]. Furthermore, other microscopic remains such as wing scales indicate the presence of moths or butterflies in the surroundings. Peaks in abundance of these fragments can be used by some authors to reconstruct insect outbreaks in ancient forests [46,47]. Apart from that, scolecodont-like palynomorphs (Fig 6I) could be associated with mandibles or even body parts of a variety of freshwater arthropods (adults and larval stages) such as ants, copepods, oribatid mites, among others [48-50].

Other palynomorphs, such as spores of bryophytes and pteridophytes, may have also been produced by parent plants growing nearby. This is the case of bryophytes (*Reboulisporites fuegiensis*, Fig 4E; *Zlivisporis* sp., Fig 4F) and ferns [e.g., (Cyatheaceae/Dicksoniaceae, *Deltoidospora minor*, Fig 4B); (Osmundaceae; *Todisporites*

*minor*, Fig 4A); (Pteridaceae, *Polypodiaceoisporites* cf. *retirugatus*, Fig 4D)]. We assume these spores have been transported by watercourses from relatively short distances. The presence of pollen grains assigned to aquatic plants such as water-plantains (Onagraceae, *Corsinipollenites menendezii*, Fig 5M) and duckweeds (Lemnoideae, Araceae; *Pandaniidites* sp., Fig 5L) also supports the occurrence of relatively quiet water bodies.

Other palynomorphs may have been produced within a short distance of the fossil site such as Osmundaceae (*Todisporites minor*, Fig 4A), Aytoniaceaae (*Rebulisporites fuegiensis*, Fig 4E), which most likely were transported to the pond from a more distant source either by insects or watercourses. Also we suggest that the pollen grains of the Phytolaccaceae (*Tricolpites membranus*, Fig 4S), Fabaceae (*Rhoipites baculatus*, plate Fig 5G), Fabaceae Caesalpinioideae (*Margocolporites tenuireticulatus*, Fig 5D), the palm family Arecaceae (*Arecipites minutiscabratus*, Fig 4K; *Verrumonocolpites* sp.), Liliaceae (*Liliacidites mirus*, Fig 4M), Ulmaceae *Phyllostylon* (*Verrustephanosporites simplex*, Fig 5O), Smilacaceae (*Smilacipites* sp. cf. *S. herbaceoides*, Fig 4J), and Malvaceae Bombacoideae (*Bombacacidites* sp., Fig 5F) may have grown near the pond. Many of these (sub)families include species with predominantly subtropical and seasonally dry modern distributions [51-53] and we suggest that they may have developed as patches near the pond, or as gallery forest along rivers. Other pollen species that became transported and preserved at the pond include the family Ephedraceae (*Equisetosporites notensis*, Fig 4I), commonly occurring throughout salt-stress and alkali-stress areas. Among non-pollen palynomorphs, fungi spores such as *Multicellites crassisporus*, Fig 6C; and *Pluricellaesporites sheffyi* (not illustrated) reinforce the hypothesis of humid conditions [40,54].

Pollen grains with clear long-distance adaptations such as those produced by southern beeches (*Nothofagidites anisoechinatus* and *N. saraensis*, Fig 4O-P) and podocarps (*Podocarpidites* sp. and *Microcachryidites antarcticus*, Fig 4G-H) may have blown in, probably from altitudinally zoned or edaphically restricted regions. The presence of these Gondwanan elements in tropical regions must not be surprising as today some of them (e.g., *Podocarpus*) coexist in the Southern Andean Yungas; a very humid narrow band of forest

between the drier Gran Chaco region to the east and the dry, high altitude Puna region to the west. Some of the Gondwanan taxa that became regionally extinct in the Puna still prevail southwards in the humid and cool Andean region of Patagonia, such as the case of the southern beech genus *Nothofagus*. ~~Als~~[illegible]he Tasmanian genus *Microcachrys* was detected in the Puna which is represented by low shrubs growing up to 1 m tall at high altitudes. It completely disappeared from South America, probably during the Miocene, after a major bloom of Gondwanan elements in Patagonia during the Oligocene [55].

A comparison with assemblages recovered from older units in northwestern Argentina (i.e., Tunal/Olmedo, Mealla, Maíz Gordo and Lumbrera formations) reveals that the assemblage from the Casa Grande 1 formation is quite different (S1 Table), particularly due to the reduced number of species in common. In fact, our cluster analysis (based on presence-absence data) shows a dramatically different hierarchical scheme with one major cluster including all Paleocene-early Eocene assemblages, all sister to the mid Eocene Casa Grande 1 Fm. as a single tip (Fig 7). Note that the stratigraphically closest Paleogene assemblages are clustered together, that is early Paleocene assemblages (Tunal/Olmedo Fm.) on one side and middle Paleocene (Mealla Fm.) and Paleocene/Eocene (Maíz Gordo Fm.) assemblages on the other. The slightly younger Lumbrera formation stands next to those assemblages in our cluster analysis.

**Fig 7. Cluster dendrogram**. Hierarchical clustering showing the similarities among the spore-pollen records from the Casa Grande 1 sequence (this study) and those previously published from other nearby Paleogene sites based on presence/absence data (S1 Table).

The differences between our palynological assemblage and those previously studied from S[illegible]lta Basin –also supported by our cluster analysis– can be either because; 1) The number of species identified in our study from the Casa Grande 1 Fm. is dramatically different from those previously reported from older deposits (taxonomic bias hypothesis); 2) The environmental conditions prevailing during the accumulation of the palynomorph bearing

level of the Casa Grande 1 Fm. favored the preservation of more spores and pollen types (taphonomic bias hypothesis); 3) The uplift of the Andean range (mid Eocene) may have driven shifts in the paleo-floras and, by extension, in the palynological assemblages (paleoecological hypothesis); 4) The increasing concentrations of atmospheric $CO_2$ during the mid-Eocene climatic optimum probably triggered shifts in plant communities (paleoclimatic hypothesis); 5) The Casa Grande Fm. would be slightly younger than previously thought and therefore new and more modern floristic components were recorded (stratigraphical hypothesis). Among these, we reject hypothesis 1 because previous palynological works (e.g., [17-19,56]) have been exhaustive, that is, they described and illustrated a large number of palynomorphs using a reasonable number of samples. In fact, the number of species identified for Casa Grande Fm. (43 species, this study) is very close to, for example, the 48 species identified for the Tunal Fm. (S1 Table). We tentatively reject hypothesis 2 because the reconstructed environmental conditions appear to have been terrestrial (i.e., ponds, swamps or shallow lakes) for all Paleogene sites from the region. We infer that hypotheses 3 and 4 are the most likely largely because of the rise of typical arid-adapted elements (e.g. Fabaceae (Mimosoideae), Poaceae and Ephedraceae), may have been driven, at least in part, by the effect of the initial stages of the Andean uplift. These families are common today in the southern Puna, and appear to have coexisted in the region since the accumulation of the Casa Grande Fm. (mid Eocene times) and onwards. On the other hand, the climatic optimum of the mid Eocene, promoted by an increasing concentration of atmospheric $CO_2$, may have favored the rise of tropical elements; for example, we identified several species of palms and other tropical-affinity lineages (e.g., Malvaceae [illegible] Bombacoideae, Smilacaceae). Finally, we cannot entirely reject hypothesis 5 because the pollen-bearing sediments of the Casa Grande Fm. can be slightly younger than mid Eocene, yet further analysis (either biostratigraphic or radiometric) is needed. However, the presence of typical species of the Paleocene-Eocene (e.g., *Verrustephanoporites simplex*) along with the complete absence of key open-habitat representatives from the

Neogene such as the widely distributed spiny pollen grains of the Asteroideae (daisy family) gives us the notion that the Casa Grande Fm. might not be younger than Paleogene.

Overall, we assume that our reconstructed mid Eocene plant community has no close analogue in the modern South American vegetation, although a broad resemblance to the seasonally-dry forest of the Chaco becomes increasingly probable. This is largely because of the co-~~occurence~~ of Fabaceae, Malvaceae Bombacoideae and Ulmaceae *Phyllostylon*, which grow today particularly across the humid and dry Chaco. Their occurrence, along with the presence of several species of palms (Arecaceae), lead us to suggest subtropical or tropical conditions and frost-free winters. More importantly, our analysis supports previous assumptions indicating that the threshold elevation at which the orographic rain shadow effect became established at these latitudes, post-date the accumulation of the Casa Grande 1 Formation.

# Acknowledgments

We thank S. Mirabelli for processing palynological samples.

PIP 666-CONICET grant to C. del Papa

# Author contributions

Analyzed the data: EEF MJT LM. Contributed materials/analysis tools: LP VDB CP. Wrote the paper: LP with inputs from EEF MJT LM VDB CP. Performed field work: CP.

# References

1. Cabrera AL. Ecología vegetal de la puna. In: Troll C, editor. Colloquium Geographicum Band 9. Proceedings of the UNESCO México Symposium. Bonn: Dümmler in Kommission; 1966. p. 91-116.
2. Matteucci SD. Ecorregión Puna. In: Morello J, Matteucci SD, Rodríguez AF, Silva M, editors. Ecorregiones y Complejos Ecosistémicos Argentinos. Buenos Aires: Orientación Gráfica SRL; 2012. pp. 87-127.
3. Pulgar Vidal J. La agricultura y la reforestación en la región Puna. Colloque d'Études Péruviennes-, Aix en Provence, pp. 273-278, Faculté de Lettres et Sciences Humaines d'Aix, France, *Aix-en-Provence, Centre d'Eludes Latino Américaines*; 1966. p. 273-279.
4. Navone SM, Maggi A, Introcaso R. Desertification, climate change and land use in Argentine Puna region since 1975. In: Winslow M, Sommer S, Bigas H, Martius C, Vogt J, Akhtar Schuster M, Thomas R, editors. Understanding Desertification and Land Degradation Trends. Proceedings of the UNCCD First Scientific Conference; 22–24 September 2009; Buenos Aires, Argentina. Luxembourg: Office for Official Publications of the European Communities; 2011. pp. 140-141.
5. Reboratti C. Situación ambiental en las ecorregiones Puna y Altos Andes. In Brown A, Martinez Ortiz U, Acerbi M, Corcuera J, editors. La situación ambiental argentina. Buenos Aires: Fundación Vida Silvestre Argentina; 2006. p. 33-39.
6. Matteucci SD. Ecorregión Puna. In Morello J, Matteucci SD, Rodríguez AF, Silva M, editors. Ecorregiones y complejos ecosistémicos argentinos. Universidad de Buenos Aires, Facultad de Arquitectura, DisenÞo y Urbanismo: GEPAMA, Grupo de Ecologiìa del Paisaje y Medio Ambiente, Universidad de Buenos Aires: Orientacioìn Grafica Editora. Buenos Aires, Argentina; 2012. p. 87-127.
7. Perovic PG, Trucco Aleman CE, Tellaeche CG, Bracamonte JC, Cuello PA, Novillo A, et al. Mamíferos puneños y altoandinos. In Grau HR, Babot MJ, Izquierdo AE, Grau A, editors. La Puna Argentina. Naturaleza y cultura. Serie Conservación de la Naturaleza, 24. Tucumán: Fundación Miguel Lillo; 2018. p. 182-206.
8. Masek JG, Isacks BL, Gubbels TL, Fielding EJ. Erosion and tectonics at the margins of continental plateaus. J Geohys Res. 1994; 99 (B7): 13941-13956.
9. Alonso RN, Bookhagen B, Carrapa B, Coutand I, Haschke M, Hilley GE, et al. Tectonics, climate, and landscape evolution of the southern central Andes: the Argentine Puna Plateau and adjacent regions between 22 and 30 S. In Oncken O,

Chong G, Franz G, Giese P, Götze HJ, Ramos VA, et al., editors. The Andes. Berlin: Springer; 2006. p. 265-283.

10. Vera C, Baez J, Douglas M, Emmanuel CB, Marengo J, Meitin J, et al. The South American low-level jet experiment. Bull Am Meteorol Soc. 2006; 87: 63–78.
11. Hyland EG, Sheldon ND, Cotton JM. Terrestrial evidence for a two-stage mid-Paleocene biotic event. Palaeogeogr Palaeoclimatol Palaeoecol. 2015; 417: 371-378.
12. Andrews E, White T, del Papa C. Paleosol-based paleoclimate reconstruction of the Paleocene-Eocene thermal maximum, northern Argentina. Palaeogeogr Palaeoclimatol Palaeoecol. 2017; 471: 181–195.
13. Do Campo M, Bauluz B, del Papa C, White T, Yuste A, Mayayo MJ. Evidence of cyclic climatic changes recorded in clay mineral assemblages from a continental Paleocene-Eocene sequence, northwestern Argentina. Sediment Geol. 2018; 368: 44-57.
14. Do Campo M, Bauluz B, Payrola P, Yuste A, Mayayo MJ. Terrestrial record of cyclic early Eocene warm-humid events in clay mineral assemblages from the Salta basin, northwestern Argentina. Sediment Geol. 2021; 425: 106004.
15. Powell JE, Babot MJ, García López DA, Deraco MV, Herrera CM. Eocene vertebrates of northwestern Argentina: annotated list. In Salfity JA, Marquillas RA, editors. Cenozoic geology of the central Andes of Argentina. Salta: SCS publisher; 2011. p. 349-370.
16. Quattrocchio M. Contribución al conocimiento de la palinología estratigráfica de la Formación Lumbrera (Terciario Inferior, Grupo Salta). Ameghiniana. 1978; 15(3-4): 285-300.
17. Quattrocchio M, Volkheimer W, del Papa C. Palynology and paleoenvironment of the “Faja Gris”; Mealla Formation (Salta Group) at Garabatal Creek (NW Argentina). Palynology. 1997; 21(1): 231-247.
18. Quattrocchio M, del Papa CE. Paleoambiente de la Secuencia Maíz Gordo (¿ Paleoceno Tardío-Eoceno Temprano?), Arroyo Las Tortugas, Cuenca del Grupo Salta (NO Argentina). Palinología y sedimentología. Spanish Journal of Palaeontology. 2000; 15(1): 57-70.
19. Volkheimer W, Novara MG, Narváez PL, Marquillas RA. Palynology and paleoenvironmental significance of the Tunal Formation (Danian) at its type locality, El Chorro creek (Salta, Argentina). Ameghiniana. 2006; 43(3): 567-584.
20. Narváez PL. Palinoestratigrafía, paleoambientes y cambios climáticos durante el Cretácico final y Paleógeno de la Cuenca del grupo Salta, República Argentina.

[Ph.D. thesis]. Mendoza: Universidad Nacional de Cuyo; 2009. Available from: https://planificacion.bdigital.uncu.edu.ar/objetos_digitales/5502/narvaez-tesisd.pdf

21. Narvaez P, Volkheimer W. Palynostratigraphy and paleoclimatic inferences of the Balbuena and Santa Bárbara subgroups (Salta Group Basin, Cretaceous-Paleogene): Correlation with Patagonian basins. In Salfity JA, Marquillas RA, editors. Cenozoic geology of the central Andes of Argentina. Salta: SCS publisher; 2011. p. 283-300.
22. Carrapa B, DeCelles PG. Eocene exhumation and basin development in the Puna of northwestern Argentina. Tectonics. 2008; 27(1).
23. López Steinmetz RL, Ávila P, Dávila FM. Landscape and drainage evolution during the Cenozoic in the Salinas Grandes Basin, Andean Plateau of NW Argentina. Geomorphology (Amst). 2020; 353: 1-16.
24. del Papa C, Hongn F, Powell J, Payrola P, Do Campo M, Strecker MR, et al. Middle Eocene-Oligocene broken foreland evolution in the Andean Calchaquí Valley, NW Argentina: insights from stratigraphic, structural and provenances studies. Basin Res. 2013; 25: 574–593.
25. Ehlers TA, Poulsen CJ. Influence of Andean uplift on climate and paleoaltimetry estimates. Earth Planet. Sci. Lett. 2009; 281(3-4): 238-248.
26. Lagomarsino LP, Condamine FL, Antonelli A, Mulch A, Davis CC. The abiotic and biotic drivers of rapid diversification in Andean bellflowers (Campanulaceae). New Phytol. 2016; 210(4): 1430-1442.
27. Montero López C, del Papa C, Hongn F, Strecker MR, Aramayo A. Synsedimentary broken- foreland tectonics during the Paleogene in the Andes of NW Argentine: new evidence from regional to centimetre- scale deformation features. Basin Res. 2018; 30: 142-159.
28. Bond M, López G. Los mamíferos de la Formación Casa Grande (Eoceno) de la provincia de Jujuy. Argentina. Ameghiniana. 1995; 32(3): 301-309.
29. Babot MJ, García López DA, Deraco V, Herrera CM, del Papa C. Mamíferos paleógenos del subtrópico de Argentina: síntesis de estudios estratigráficos, cronológicos y taxonómicos. In Muruaga CM, Grosse P, editors. Ciencias de la Tierra y Recursos Naturales del NOA: Relatorio del XX Congreso Geológico Argentino; 2017 August 7-11; Tucumán, Argentina. Tucumán. 2017. p. 730-753.
30. Fernández J, Bondesio P, Pascual R. Restos de Lepidosiren paradoxa (Osteichthyes, Dipnoi) de la Formación Lumbrera (Eógeno, ¿Eoceno?) de Jujuy. Ameghiniana. 1973; 10(2): 152-172.
31. Boll A. Hernández RM. Interpretación estructural del área Tres Cruces: Boletín de Informaciones Petroleras. Tercera Época. 1986; 3(7): 2-14.

32. DeCelles PG, Carrapa B, Horton BK, Gehrels GE. Cenozoic foreland basin system in the central Andes of northwestern Argentina: Implications for Andean geodynamics and modes of deformation. Tectonics. 2011; 30(6): 1-30.
33. Del Papa C, Kirschbaum A, Powell J, Brod A, Hongn F, Pimentel M. Sedimentological, geochemical and paleontological insights applied to continental omission surfaces: a new approach for reconstructing an Eocene foreland basin in NW Argentina. J South Am Earth Sci. 2010; 29(2): 327-345.
34. Herrera CM, Powell JE, del Papa CE. Un nuevo Dasypodidae (Mammalia, Xenarthra) de la Formación Casa Grande (Eoceno) de la provincia de Jujuy, Argentina. Ameghiniana. 2012; 49(2): 267-271.
35. Phipps D, Playford G. Laboratory techniques for extraction of palynomorphs from sediments. Papers. Department of Geology. University of Queensland. 1984; 11(1): 1-23.
36. Traverse A. Topics in geobiology. Paleopalynology. 2nd ed. Dordrecht, the Netherlands: Springer; 2007.
37. Noetinger S, Pujana RR, Burrieza A, Burrieza HP. Use of UV-curable acrylates gels as mounting media for palynological samples. Revista del Museo Argentino de Ciencias Naturales. 2017; 19(1): 19-23.
38. Punt W, Hoen PP, Blackmore S, Nilsson S, Le Thomas A. Glossary of pollen and spore terminology. Rev. Palaeobot. Palynol. 2007; 143(1-2): 1-81.
39. R Core Team. R: A language and environment for statistical computing. R Foundation for Statistical Computing, Vienna, Austria. 2019; URL https://www.R-project.org/.
40. Kalgutkar RM, Braman DR. Santonian to? earliest Campanian (Late Cretaceous) fungi from the Milk River Formation, Southern Alberta, Canada. Palynology. 2008; 32(1): 39-61.
41. Van Geel, B. Non-pollen palynomorphs. In Smol JP, Birks HJB, Last WM, editors. Tracking environmental change using lake sediments Dordrecht: Springer; 2002. Vol. 3. Terrestrial, Algal, and Siliceous Indicators. p. 99-119.
42. Cook EJ, Van Geel B, Van der Kaars S, Van Arkel, J. A review of the use of non-pollen palynomorphs in palaeoecology with examples from Australia. Palynology. 2011; 35(2): 155-178.
43. Weigmann G, Deichsel R. Acari: limnic oribatida. In Gerecke R, editor. Süßwasserfauna von Mitteleuropa. Berlin: Springer; 2006 (Bartsch I, Davids K, Deichsel R, Di Sabatino A, Gabrys G, Gledhill T, et al. Chelicerata: Araneae/Acari I Vol. 7/2-1). p 89-112.

44. Hall SA. Atmospheric transport of freshwater algae Pediastrum in the American Southwest. Grana. 1998; 37: 374-375.
45. Kołaczek P, Karpińska Kołaczek M, Worobiec E, Heise W. Debarya glyptosperma (De Bary) Wittrock 1872 (Zygnemataceae, Chlorophyta) as a possible airborne alga: a contribution to its palaeoecological interpretation. Acta Palaeobotanica. 2012; 52(1): 139-146.
46. Montoro Girona M, Navarro L, Morin H. A secret hidden in the sediments: Lepidoptera scales. Front. Ecol. Evol. 2018; 6: 1-5.
47. Navarro L, Harvey AÉ, Morin H. Lepidoptera wing scales: a new paleoecological indicator for reconstructing spruce budworm abundance. Can J For Res. 2018; 48(3): 302-308.
48. Van Geel B. A palaeoecological study of Holocene peat bog sections in Germany and the Netherlands, based on the analysis of pollen, spores and macro-and microscopic remains of fungi, algae, cormophytes and animals. Rev Palaeobot Palynol.1978; 25(1): 1-120.
49. Van Geel B, Coope GR, Van Der Hammen T. Palaeoecology and stratigraphy of the Lateglacial type section at Usselo (The Netherlands). Rev Palaeobot Palynol. 1989; 60(1-2): 25-129.
50. Shumilovskikh L, O'Keefe JM, Marret F. An overview of the taxonomic groups of non-pollen palynomorphs. The Geological Society Special Publications. 2021; 511(1): 13-61.
51. Nielsen IC. Mimosaceae (Leguminosae-Mimosoideae). Flora Malesiana - Series 1, Spermatophyta. 1992; 11(1): 1–226.
52. Pennington RT, Prado DE, Pendry CA. Neotropical seasonally dry forests and Quaternary vegetation changes. J Biogeogr. 2000; 27(2): 261-273.
53. Zizka A, Carvalho Sobrinho JG, Pennington RT, Queiroz LP, Alcantara S, Baum DA, et al. Transitions between biomes are common and directional in Bombacoideae (Malvaceae). J Biogeogr. 2020; 47(6): 1310-1321.
54. Premaor E, Saxena RK, de Souza PA, Kalkreuth W. Fungal spores and fruiting bodies from Miocene deposits of the Pelotas Basin, Brazil. Revue de micropaléontologie. 2018; 61(3-4): 255-270.
55. Barreda VD, Palazzesi L, Pujana RR, Panti C, Tapia MJ, Fernández DA, Noetinger S. The gondwanan heritage of the eocene–miocene patagonian floras. J South Am Earth Sci. 2021; 107:103022.
56. Quattrocchio ME, Volkheimer W. Paleogene paleoenvironmental trends as reflected by palynological assemblage types, Salta Basin, NW Argentina. Neues Jahrb Geol Palaontol Abh. 1990: 377-396.

# Supporting information

**S1 Table. Data matrix (presence/absence) of pollen and spores from the Paleogene of the Salta Group.** OF: Olmedo Formation, TF: Tunal Formation, MF: Mealla Formation, MGF: Maíz Gordo Formation, LF: Lumbrera Formation, CGF: Casa Grande Formation (this work).

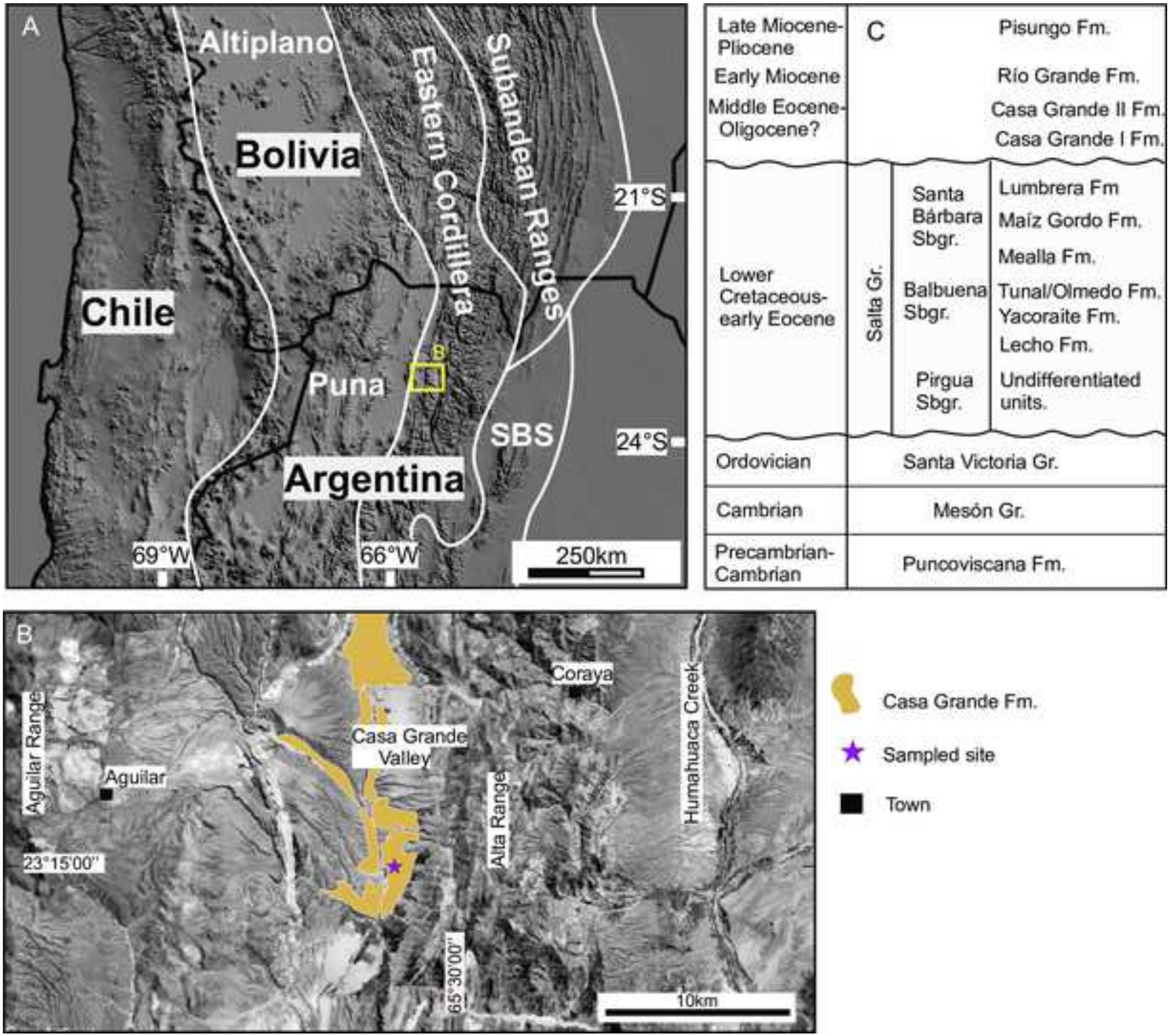
A
Altiplano
Bolivia
Chile
Eastern Cordillera
Subandean Ranges
21°S
Puna
B
SBS
24°S
Argentina
69°W
66°W
250km
C
Late Miocene-Pliocene
Pisungo Fm.
Early Miocene
Río Grande Fm.
Middle Eocene-Oligocene?
Casa Grande II Fm.
Casa Grande I Fm.
Lower Cretaceous-early Eocene
Salta Gr.
Santa Bárbara Sbgr.
Lumbrera Fm
Maíz Gordo Fm.
Mealla Fm.
Balbuena Sbgr.
Tunal/Olmedo Fm.
Yacoraite Fm.
Lecho Fm.
Pirgua Sbgr.
Undifferentiated units.
Ordovician
Santa Victoria Gr.
Cambrian
Mesón Gr.
Precambrian-Cambrian
Puncoviscana Fm.
B
Aguilar Range
Aguilar
23°15'00"
Casa Grande Valley
Coraya
Alta Range
Humahuaca Creek
65°30'00"
10km
Casa Grande Fm.
Sampled site
Town

Figure 2

Click here to access/download;Figure;Fig2.tif

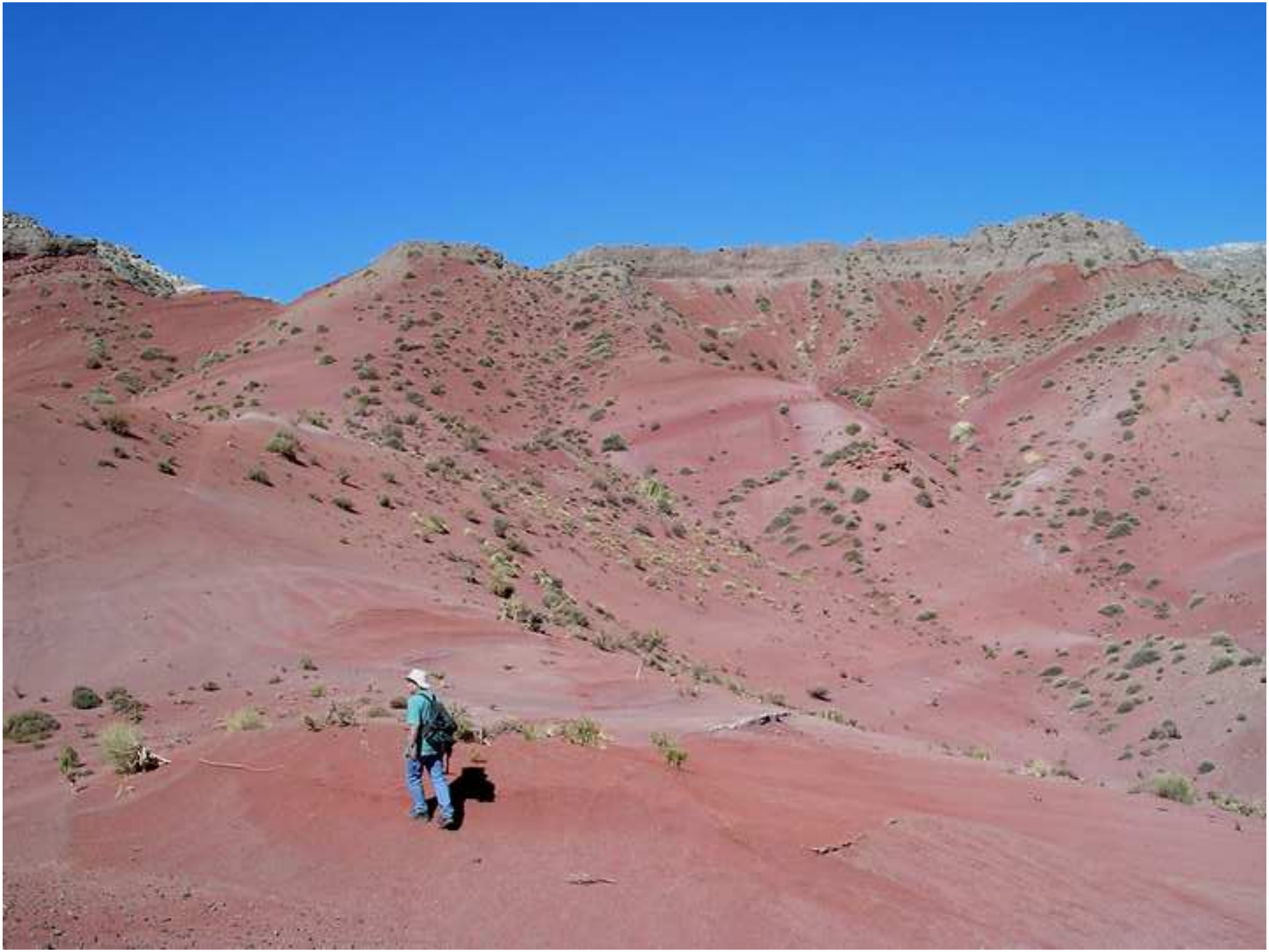

Figure 3

Click here to access/download;Figure;Fig3.tif

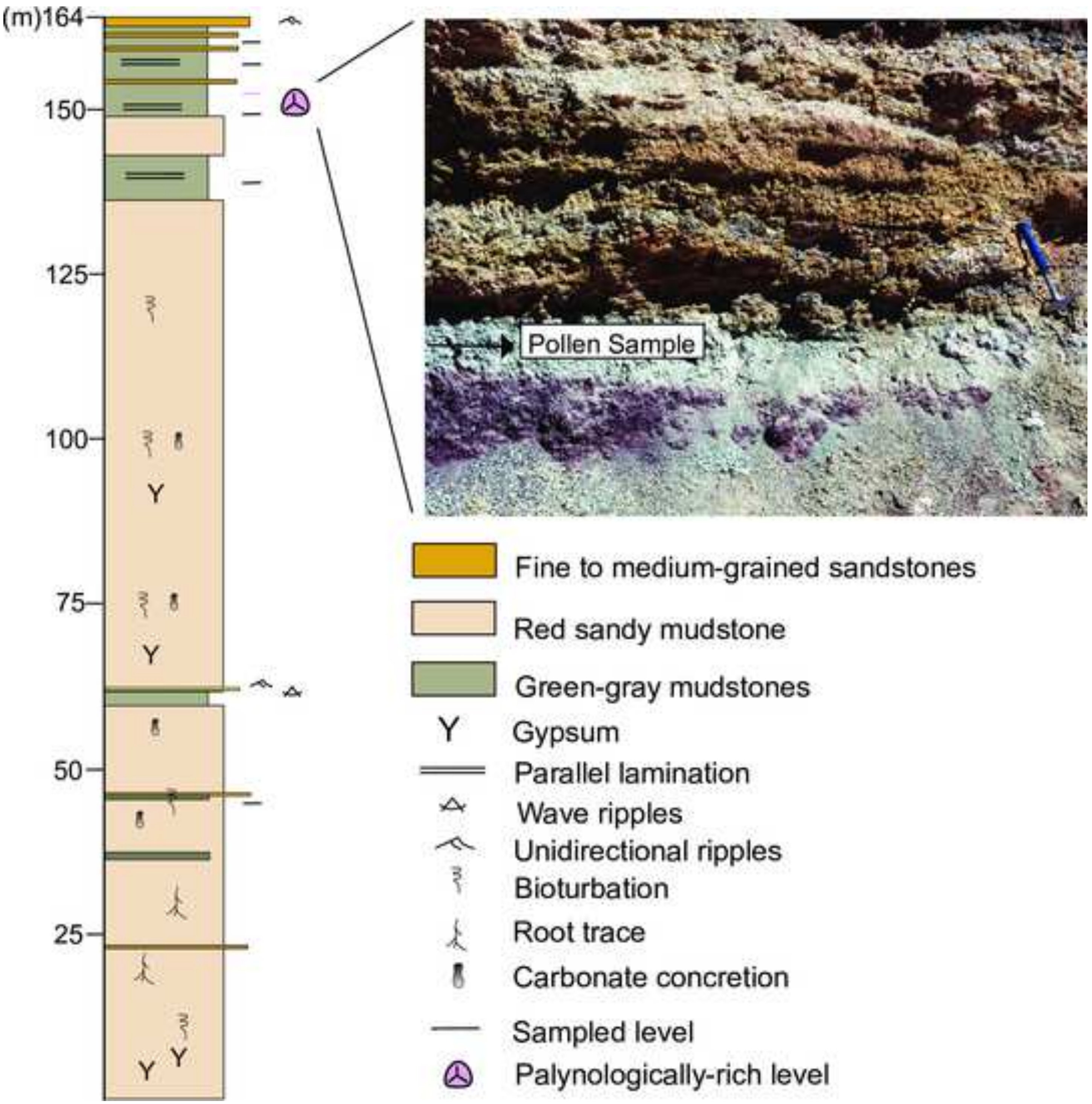

 Click here to access/download;Figure;Fig4.tif

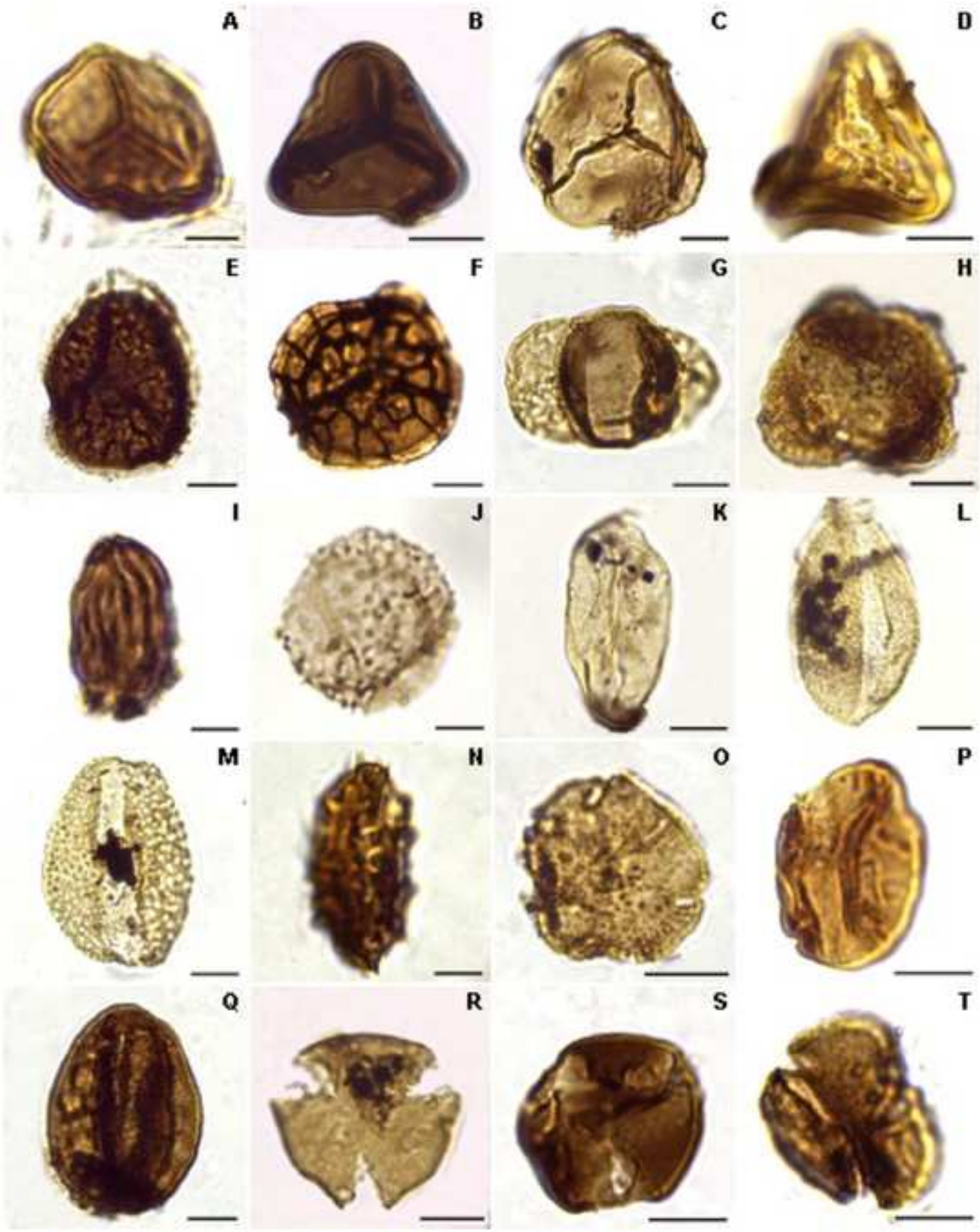

Figure 5

Click here to access/download;Figure;Fig5.tif

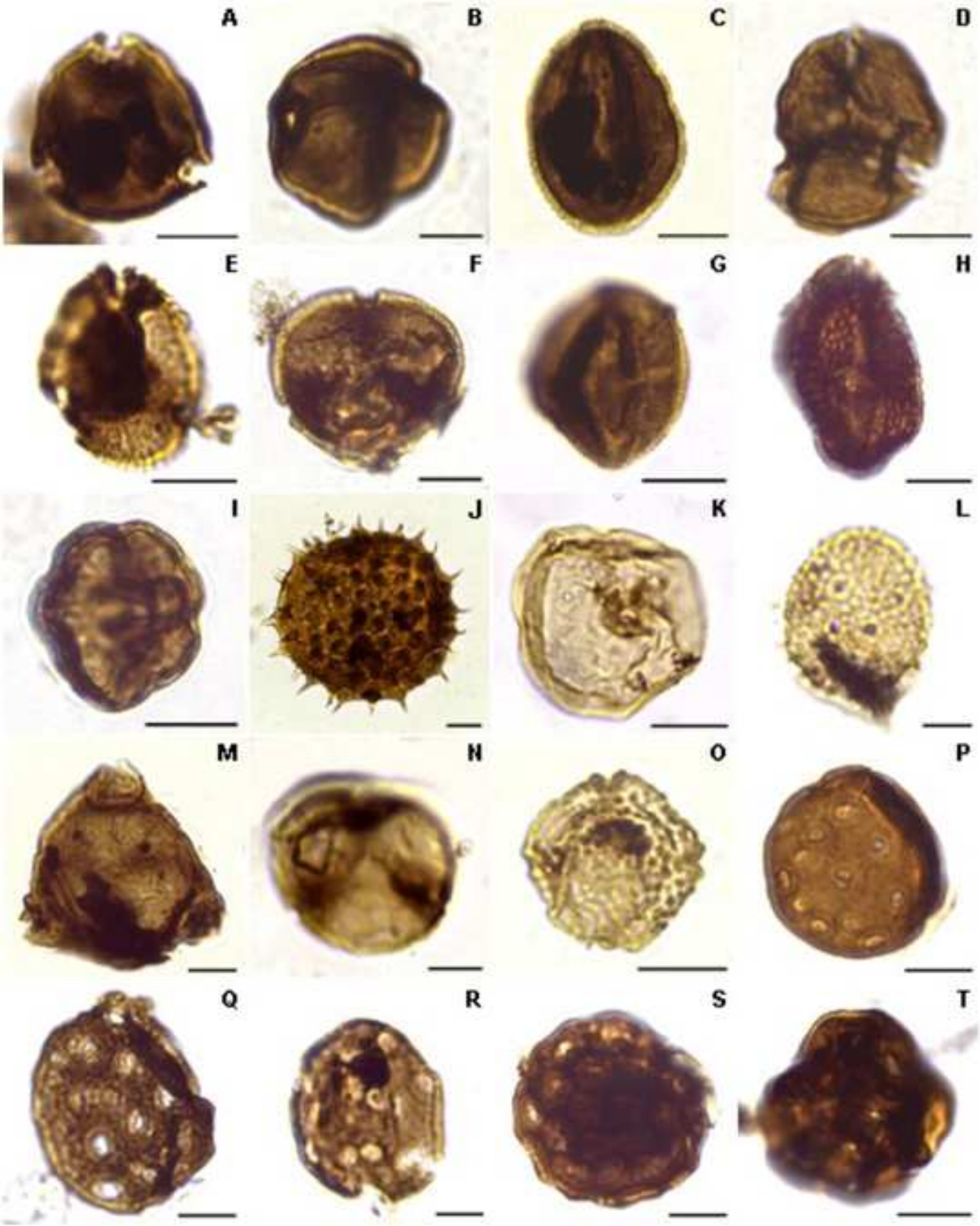

Figure 6

Click here to access/download;Figure;Fig6.tif

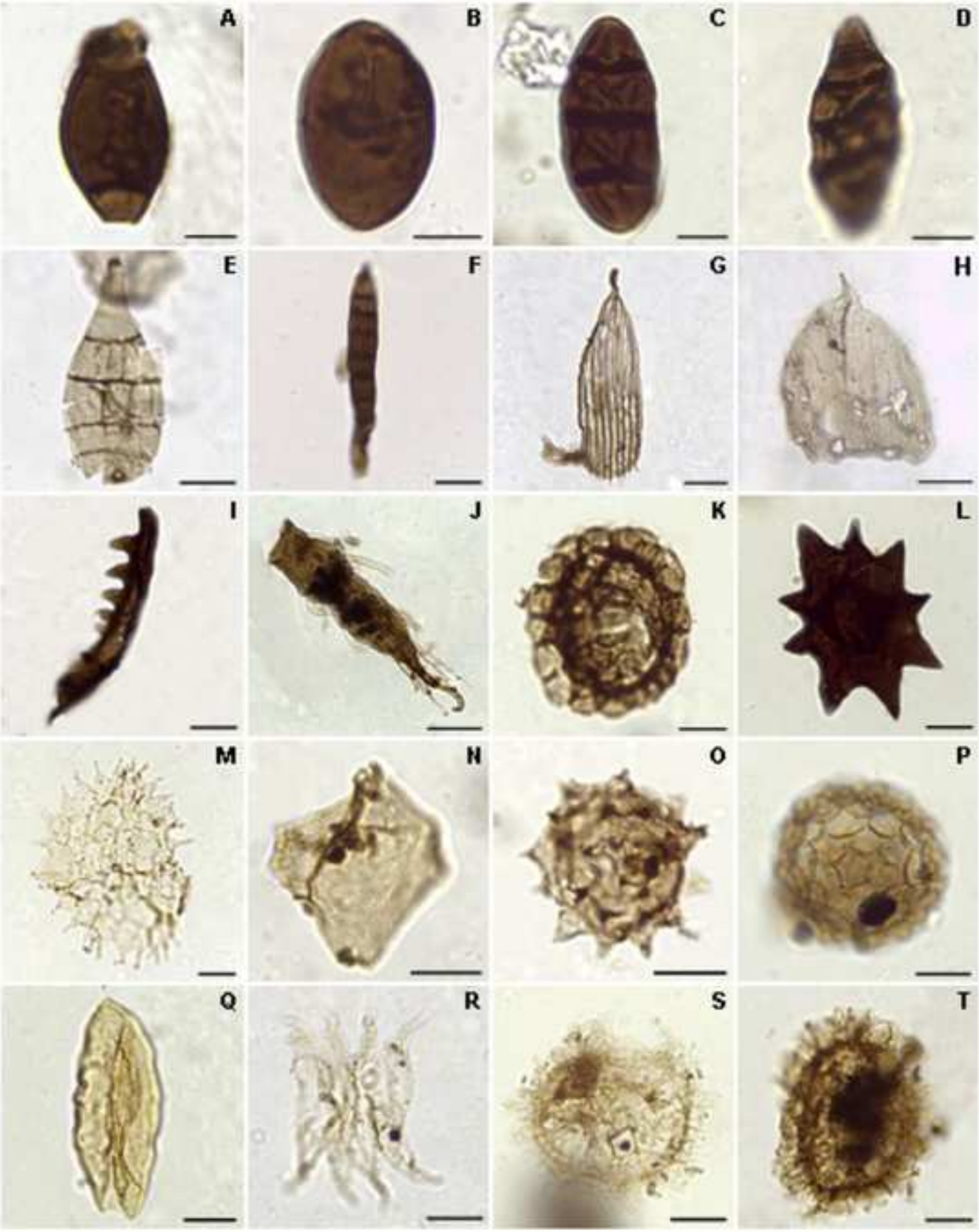

Figure 7

Click here to access/download;Figure;Fig7.tif

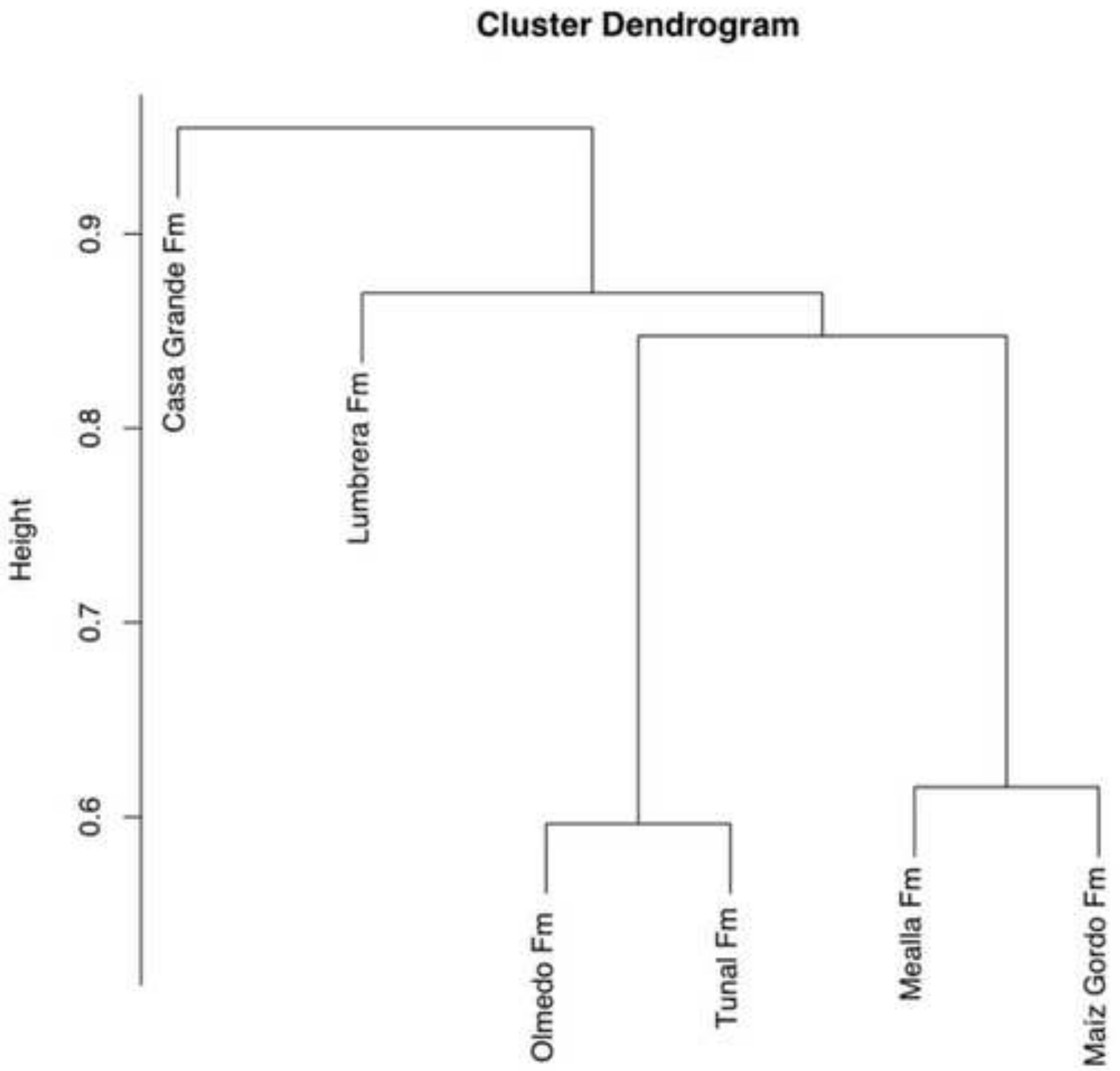

Supporting Table 1

Click here to access/download
**Supporting Information**
S1 Table.docx
