# Supplementary material for: A snapshot of mid Eocene landscapes in the southern Central Andes: Spore-pollen records from the Casa Grande Formation (Jujuy, Argentina)
Source: PLoS One. 2023 Apr 5;18(4):e0277389. doi: 10.1371/journal.pone.0277389 (PMC10075436; doi:10.1371/journal.pone.0277389)
Supplement: Attachment — Submitted filename: Response to Reviewers.docx [file pone.0277389.s007.docx]

Buenos Aires, January 19^th^ 2023

Dear Dr. Gongle Shi,

Below, a point-by-point response is provided in red to all requests given by the Reviewers. The manuscript has now been improved by incorporating the reviewer's comments; please, thank them on our behalf.

Reviewer #1: Farrell et al. documented the mid-Eocene floristic composition of the Casa Grande Formation in the Puna region for the first time, and reconstructed its landscape. The authors found about 70 palynomorphs including a few Gondwanan taxa, forming a frost-free and humid to seasonally dry ecosystem near a lacustrine environment. This is a preliminary study with only one analyzable pollen sample among seven collected samples, and there has some space for improvement.

1. The hierarchical clustering cannot be used for comparison in this study, because this research has only one sample, while others have some samples (e.g., In ref. 16, it has five samples. In ref. 17, it has four samples? In ref. 19, it has 16 (of 26 samples) yielding palynomorphs. In ref. 20, it has 5 (of 9 samples) yielding palynomorphs). This is sampling bias which the authors did not discuss in the text. The authors will have to revise/delete corresponding text.

Response #1: We appreciate Reviewer #1 comments. In the new version, we have discussed in the main manuscript (lines 146-148) the sampling bias that our analysis faced. We are aware that the comparison of sedimentary sections with different sampling efforts may cause misleading results. However, the number of palynomorphs recovered, which is the main source of comparisons, is relatively similar among sections. We have now included the number of samples and the number of palynomorphs used for the hierarchical clustering (lines 149-151, S3 Table). And we have now explicitly expressed that results should be interpreted with caution.

2. As the Casa Grande assemblage is quite different from those produced from older sediments, it is useful for the authors to add a systematics section to describe some important taxa.

Response #2: The new version of our manuscript now includes a systematic section (Supplement 1 Appendix) to describe and/or comment on some key species.

3. For the reconstructed landscape, the authors can draw a landscape model for a better visualization, and maybe also for the five compared localities.

Response #3: We have now included a reconstructed landscape based on the interpretations of the botanical affinity of the discovered palynomorphs from the Casa Grande Formation (Fig. 8). The reconstruction of landscapes from other Paleogene sites may be an interesting feature to include, although it goes beyond the main goal of our work.

4. For the other comments, please refer to the annotated PDF.

Response #4: We included all modifications suggested by Reviewer #1 in this new version of the manuscript. We appreciate Reviewer #1 corrections and suggestions; please, thank him/her on our behalf.

Reviewer #2: The hierarchical clustering is lack of percentage values, instead, only depends on the absence/presence data, that seems not convictive. I suggest publication after providing the pollen counting data and hope my comments are helpful.

Other minor comments please refer to the attached comments in the pdf.

Response #5: We have now provided a pollen-counting dataset (S2 Table). We used presence/absence data for our hierarchical clustering analysis because not all publications provided pollen-counting data, but a list of species, instead. We have now specified this in the methods, and commented on the limitations our approach faced.

We have also included a new (S3 Table) with the sedimentary units used for comparison along with the number of samples and species that each of them includes. We appreciate all the comments of Reviewer #2 from the pdf, which improved the quality of our work. Please, thank him/her on our behalf.
